# Differences in the risk association of TERT-CLPTM1L rs4975616 (A>G) with lung cancer between Caucasian and Asian populations: A meta-analysis

**DOI:** 10.1371/journal.pone.0309747

**Published:** 2024-09-10

**Authors:** Xiaozheng Wu, Wen Li, Yunzhi Chen

**Affiliations:** Department of Preclinical medicine, Guizhou University of Traditional Chinese Medicine, Guiyang, Guizhou, China; Tabriz University of Medical Sciences, ISLAMIC REPUBLIC OF IRAN

## Abstract

**Background:**

Although the G allele variant of TERT-CLPTM1L rs4975616 has been confirmed to be negatively associated to the risk of lung cancer (LC), some other studies haven’t found this negative association. The purpose of this study is to clarify the association of the rs4975616 with the risk of developing LC and the differences of this association among patients with different ethnicities (Caucasians and Asians), different subtypes of LC, and different smoking status.

**Methods:**

Relevant literatures published before July 20, 2023 in PubMed, EMbase, Web of Science, MEDLINE databases were searched through the Internet. Statistical analysis of data was performed in Revman5.3, including drawing forest plots, funnel plots and so on. Sensitivity and publication bias were performed in Stata 14.0. The stability of the results was assessed using Test Sequence Analysis (TSA) software. Registration number: CRD42024568348.

**Results:**

The G allele variant of rs4975616 was negatively associated with the risk of LC ([OR] = 0.86, 95%CI [0.84, 0.88]), and that this negative association was present in both Caucasians ([OR] = 0.85, 95%CI [0.83, 0.87]) and Asians ([OR] = 0.91, 95%CI [0.86, 0.95]), and the strength of the negative association was higher in Caucasians than in Asians (subgroup differences: P = 0.02, I^2^ = 80.3%). Across LC subtypes, rs4975616[G] was negatively associated with the risk of NSCLC (LUAD, LUSC) in both Caucasians and Asians (P<0.05) and the strength of the association with NSCLC (LUAD) was higher in Caucasians than in Asians (Subgroup differences: I^2^>50%). In Caucasians, rs4975616[G] was negatively associated with the risk of LC in both smokers and non-smokers (P<0.05), and the strength of the association did not differ between smokers and non-smokers (Subgroup differences: P = 0.18, I^2^ = 45.0%). In Asians, rs4975616[G] was mainly negatively associated with the risk of LC in smokers (P<0.05) but not in non-smokers ([OR] = 0.97, 95%CI [0.78, 1.20]). Comparisons between the two populations showed that the strength of this negative association was higher in Caucasian non-smokers than in Asian non-smokers (Subgroup differences: P = 0.04, I^2^ = 75.3%), whereas the strength of this negative association was the same for Caucasian smokers as for Asian smokers (Subgroup differences: P = 0.42, I^2^ = 0%). Among the different LC subtypes, rs4975616[G] was negatively associated with the risk of NSCLC (LUAD) incidence in both Asian smokers and Caucasian non-smokers (P<0.05), whereas it was not associated with the risk of NSCLC development in Asian non-smokers (P>0.05). Comparisons between the two populations showed that the strength of the association was higher in Caucasian non-smokers than in Asian non-smokers (Subgroup differences: I^2^>50%).

**Conclusion:**

The G allele variant of rs4975616 is negatively associated with the risk of LC and NSCLC (LUAD, LUSC). Compared with Asians, Caucasians are more likely to have a higher risk of LC and NSCLC (LUAD) due to the rs4975616 variant. In Caucasians, smoking and other factors like non-smoking contribute to rs4975616 variations leading to LC, and other factors like non-smoking also induce rs4975616 variations leading to NSCLC (LUAD). In Asians, smoking is the major risk factor for the induction of rs4975616 variations leading to LC and NSCLC(LUAD).

## 1. Introduction

Lung cancer (LC) was one of the cancers with a high mortality rate in the world, accounting for about one quarter of all cancer deaths [1]. Smoking had been recognized as a major risk factor for the development of LC [2]. However, not only smoking, but also genetic variability was an important cause of LC. Previous genome-wide association studies (GWAS) in various populations had identified dozens of risk loci for LC [3, 4], and most of these loci were clustered in the TERT-CLPTM1L region of chromosome 5p15.33 [5–11].

Cleft lip and cleft palate transmembrane protein 1 (CLPTM1L, alias CRR9) was responsible for encoding cleft lip and palate-associated transmembrane 1-like proteins. Over-expression of CLPTM1L had been observed in LC cells, and its over-expression promoted LC cell growth and survival and was required for KRAS (kirsten rat sarcoma viral oncogene)-driven LC [12, 13]. However, the function of CLPTM1L and its role in LC remain unclear. Another study had already reported that CLPTM1L, an anti-apoptotic factor commonly over-expressed in LC, protected cells from genotoxic stress-induced apoptosis by regulating Bcl-xL, suggesting its inhibitory role in genotoxic stress-induced apoptosis [13]. Later studies had also already reported that CLPTM1L was over-expressed in human ovarian tumor cell lines and was resistant to cisplatin-induced apoptosis [14–16]. All these evidences suggested that it had anti-apoptotic function on tumor cells. In addition, CLPTM1L would function not only in relation to its own biological activity, but also because it was in a region of high linkage disequilibrium (LD) with the telomerase reverse transcriptase (TERT): the entire CLPTM1L gene was located in a 62 kb LD region that contained the promoter at the 5’ end of TERT [17]. Genetic polymorphisms in TERT had been reported to associate with telomere length [18–20], and longer telomere length contributed to an increased risk of LC [21–23]. And genetic polymorphisms in CLPTM1L were associated with shorter telomere length [24], suggesting that CLPTM1L would be involved in telomere biology and even LC development together with TERT. Thus, the polymorphisms in CLPTM1L may be in cascade disequilibrium with some causal motifs in the TERT promoter, but these motifs have not been characterized so far.

The variant rs4975616 (A>G) located in the TERT-CLPTM1L region had been found to be associated with the risk of developing LC in a previous large GWAS [25]. Several later studies in Caucasian populations [11, 26, 27] and Asian populations [28, 29] had also found the significant association with the risk of developing LC. These studies collectively showed that the minor G allele variant of rs4975616 was negatively associated with the risk of LC, meaning that its minor G allele frequency was reduced in LC patients compared to healthy individuals, which implies that the G allele variant reduces the risk of developing LC. On the contrary, the major A allele variant of rs4975616 was positively associated with the risk of LC development, which indicates that the A allele is a risk allele for LC development. However, several other studies in Asian populations did not find the G allele of rs4975616 to be negatively associated with the risk of developing LC [30–34]. The study by Wang et al. (Texas-GWA) also did not find the association between rs4975616 and the risk of NSCLC incidence in a white American population [25]. The reasons for these different results may be related to different ethnicities, countries, study methods, sample sizes, types of LC, smoking status of LC patients, and patterns of genetic linkage disequilibrium. In addition, no meta-analysis has been conducted to investigate the association between rs4975616 variants and the risk of developing LC. Therefore, the results of the association between rs4975616 polymorphisms and the risk of LC development currently lack a unified and definitive conclusion.

The present meta-analysis included data from genome-wide association studies and case-control studies reporting the association of the TERT-CLPTM1L rs4975616 (A >G) polymorphism with LC to date, with the aim of clarifying the association of the rs4975616 polymorphism with the risk of developing LC and the differences of this association among patients with different ethnicities (Caucasians and Asians), different subtypes of LC, and different smoking status.

## 2. Data and methods

This study has been registered in PROSPERO(https://www.crd.york.ac.uk/prospero/), registration number: CRD42024568348.

### 2.1 Inclusion and exclusion criteria

#### 2.1.1 Inclusion criteria

① The type of studies should be genome-wide association studies (GWAS) or case-control studies of the TERT-CLPTM1L rs4975616 (A>G) polymorphism and susceptibility to LC. The language of these studies should be English, the ethnic groups should be Caucasians or Asians, and the assay for the gene should be accurately described; ② Allele frequency data were used to calculate Odds ratios (OR) and 95% confidence intervals (95% CI); ③The distribution of genotype frequency of controls conforms to Hardy-Weinberg (HWE) [35]; ④The score of Newcastle Ottawa scale (NOS) [36] should be no less than 7(≧7).

#### 2.1.2 Exclusion criteria

① Studies without allele data; ② Studies of the types of reviews, meta-analyses, conference reports and case reports; ③ Studies with pedigree as the reporting object; ④ Same studies have published for multiple times, only the one with the most complete data will be included.

### 2.2 Outcomes

The pre-specified primary outcomes were to investigate whether the rs4975616 (A > G) variant was associated with LC risk in the overall populations. The secondary outcomes were to determine whether there were differences in the strength of the association between the two populations for rs4975616 (A > G) and LC (including the various subtypes).

### 2.3 Retrieval strategy

Relevant literatures in PubMed, EMbase, Web of Science, MEDLINE databases published before July 20, 2023 were searched by theme words and keywords. The language was limited to English. Search terms (S1A Table): “Lung cancer” OR “LC” AND “rs4975616” OR “CLPTM1L” OR “TRET” AND “polymorphism”. At the same time, manual retrieval and literature tracing methods were also used to expand the search scope.

### 2.4 Literature screening and data extraction

Two relatively independent researchers (X-ZW and WL) completed literature searching and screening according to the inclusion criteria, and they cross checked and discussed them afterwards. For the literatures with different opinions, the third party (Y-ZC) made the decision. For some literatures with incomplete data, they tried to contact the author by e-mail to obtain the complete data. Finally, data extraction was carried out for the literatures being chosen after the final decision. These data include: author, year of publication, country, ethnicity, smoking status of subjects, type of LC, number of cases in case and control groups, frequency of each genotype in case and control groups, and the OR and 95% CI of each genotype.

### 2.5 Literature quality evaluation

The quality of the included literature was evaluated in the NOS [36] (X-ZW and WL), and those with a score of no less than 7 were considered as literatures with high-quality.

### 2.6 Statistical methods

The HWE of the genotypes of the controls was detected by Pearson’s chi-square test in SPSS 24.0. All results were statistically counted and analyzed in Revman 5.3, including drawing forest plots and funnel plots. Because the included eligible studies were conducted in genetically diverse populations, all results of this meta-analysis were statistically analyzed using the random effects models. The effect size and effect value of the statistical results were presented by OR value and 95% CI.

We used Q-tests to test for heterogeneity, which was quantified by I^2^. When P>0.1 or I^2^<50% indicated that there was no significant heterogeneity in all studies or in all subgroups, and the Comprehensive Meta-Analysis (CMA) v4 software was used to calculate the 95% Prediction Interval (95% PI) to assess the dispersion of effects across included studies. Meta-regression using any relevant clinical or biological characteristics of LC patients as independent variables was performed to further explore sources of heterogeneity. Begg’s Test and Egger’s Test were performed in Stata 14.0 to assess publication bias among studies, and sensitivity analysis was performed to assess the results of statistical analysis with greater heterogeneity. TSA 0.9.5.10 software was performed for the TSA tests to evaluate the stability of the conclusion ([Type I error] probability = 5%, statistical test power = 80%, relative risk reduction = 20%).

### 2.7 Ethics and dissemination

This review does not require ethical approval because the included studies are published data and do not involve the patients’ privacy. The results of this review will be reported in accordance with the PRISMA extension statement and disseminated to a peer-reviewed journal.

## 3. Results

### 3.1 Literature search results

**Fig 1:**
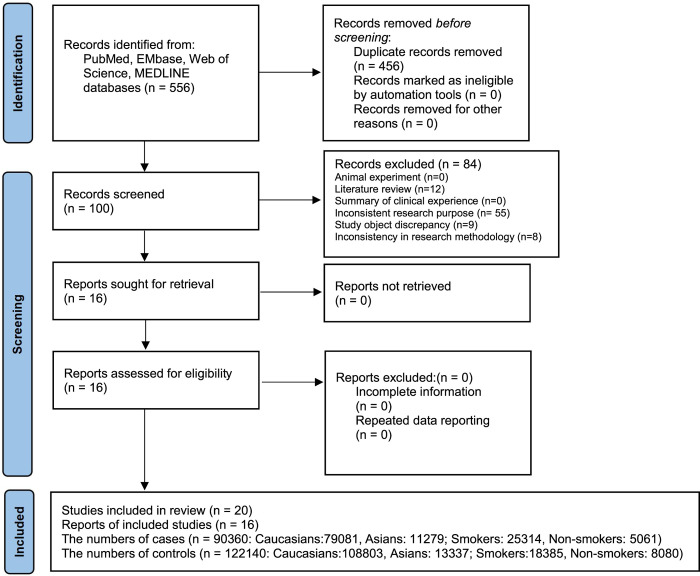
PRISMA literature screening flow diagram.

4 databases initially examined 556 literatures. After screening, 20 studies of 16 literatures were finally included (S1B Table), including 12 studies of Caucasians [5, 11, 25–27, 37–40], 8 studies of Asians [28–34, 40] (S2 and S3 Tables). These studies included 90360 LC patients and 122140 healthy controls, including 79081 patients in Caucasians and 11279 patients in Asians, and included 25314 smoking and 5061 non-smoking LC patients. The flow diagram was made according to the PRISMA statement (Fig 1).

### 3.2 Quality evaluation

All 20 studies had high NOS [36] assessment scores (≥ 7), indicating that they were all at low risk of bias (S4 Table).

### 3.3 Meta-analysis

**Fig 2:**
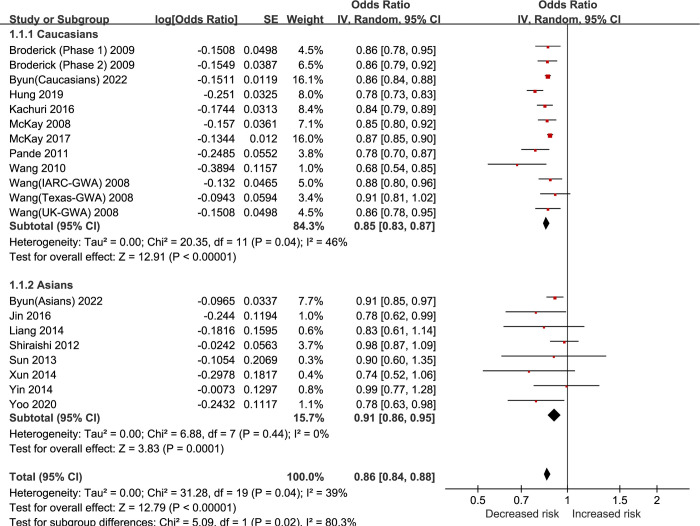
Forest plot of rs4975616 (G vs. A) for LC.

The results showed that the G allele variant of rs4975616 (allelic model) was negatively associated with the risk of developing LC in the overall population ([OR] = 0.86, 95%CI [0.84, 0.88]), and that this negative association was present in both Caucasian populations ([OR] = 0.85, 95%CI [0.83, 0.87]) and in Asian populations ([OR] = 0.91, 95%CI [0.86, 0.95]), and the strength of the negative association was higher in Caucasians than in Asians (Caucasians: [OR] = 0.85 /Asians: [OR] = 0.91, subgroup differences: P = 0.02, I^2^ = 80.3%) (Table 1, Fig 2). In additive (GG vs. AA), heterozygous (GA vs. AA), dominant (GG+GA vs. AA), and recessive (GG vs. GA+AA) models, the variant of rs4975616 was negatively associated with the risk of developing LC in the overall population (GG vs. AA: [OR] = 0.74, 95%CI [0.66, 0.83]; GA vs. AA: [OR] = 0.86, 95%CI [0.80, 0.93]; GG+GA vs. AA: [OR] = 0.84, 95%CI [0.77, 0.91]; GG vs. GA+AA: [OR] = 0.81, 95%CI [0.73, 0.90]), and these negative associations were predominantly found in Caucasian populations (p < 0.05) rather than in Asian populations (P > 0.05) (S5 Table and S1–S4 Figs).

**Table 1:** The results of meta-analysis and publication bias (rs4975616: Allele genetic model, G vs. A).

LC subtypes	Subgroups	Studies (n)	Heterogeneity test		Sample		Model	OR[95% CI]	Effect p value	Subgroup differences		Publication bias		OR[95% PI]
			P values	I^2^(%)	Cases (n)	Controls (n)				P values	I^2^(%)	P_Begg_	P_Egger_	
**LC**	Caucasians	12	0.04	46	158162	217606	Random	**0.85 [0.83, 0.87]**	**<0.00001**	**——**	**——**	0.732	0.113	0.85 [0.80, 0.91]
	Asians	8	0.44	0	22558	26674	Random	**0.91 [0.86, 0.95]**	**0.0001**	**——**	**——**	0.805	0.235	0.91 [0.86, 0.95]
	Overall	20	0.04	39	180720	244280	Random	**0.86 [0.84, 0.88]**	**<0.00001**	**0.02**	**80.3**	0.675	0.031	0.86 [0.80, 0.91]
**NSCLC** ^**a**^	Caucasians	8	0.19	30	78174	341156	Random	**0.85 [0.83, 0.87]**	**<0.00001**	**——**	**——**	0.216	0.154	0.85 [0.81, 0.89]
	Asians	7	0.43	0	17480	36014	Random	**0.90 [0.86, 0.95]**	**0.0001**	**——**	**——**	0.453	0.471	0.90 [0.86, 0.95]
	Overall	15	0.12	32	95654	377170	Random	**0.85 [0.83, 0.88]**	**<0.00001**	**0.03**	**78.4**	0.152	0.089	0.86 [0.81, 0.90]
**SCLC**	Overall	2	0.54	0	314	2312	Random	0.91 [0.68, 1.23]	0.54	**——**	**——**	——	——	0.91 [0.68, 1.23]
**LUAD** ^**b**^	Caucasians	4	0.005	77	46032	169850	Random	**0.83 [0.78, 0.88]**	**<0.00001**	**——**	**——**	0.174	0.014	0.83 [0.66, 1.05]
	Asians	5	0.36	8	14088	24064	Random	**0.92 [0.86, 0.98]**	**0.01**	**——**	**——**	0.624	0.827	0.92 [0.80, 1.05]
	Overall	9	0.004	64	61020	193914	Random	**0.86 [0.82, 0.90]**	**<0.00001**	**0.03**	**79.8**	0.233	0.000	0.86 [0.75, 0.97]
**LUSC**	Caucasians	4	0.97	0	29754	170138	Random	**0.84 [0.82, 0.87]**	**<0.00001**	**——**	**——**	0.497	0.578	0.84 [0.82, 0.87]
	Asians	3	0.75	0	3392	12350	Random	**0.86 [0.77, 0.96]**	**0.006**	**——**	**——**	0.602	0.628	0.86 [0.77, 0.96]
	Overall	7	0.99	0	33146	182488	Random	**0.84 [0.82, 0.87]**	**<0.00001**	0.75	0	0.393	0.383	0.84 [0.82, 0.87]

LC: Lung cancer; NSCLC: non-small-cell lung carcinoma; LUAD: Lung adenocarcinoma; LUSC: Lung squamous cell carcinoma.

a: NSCLC(Overall) vs. SCLC(Overall): Test for subgroup differences: Chi^2^ = 0.18, df = 1 (P = 0.67), I^2^ = 0%.

b: LUAD(Overall) vs. LUSC(Overall): Test for subgroup differences: Chi^2^ = 1.18, df = 1 (P = 0.61), I^2^ = 0%; LUAD(Caucasians) vs. LUSC(Caucasians): Test for subgroup differences: Chi^2^ = 0.29, df = 1 (P = 0.59), I^2^ = 0%

LUAD(Asians) vs. LUSC(Asians): Test for subgroup differences: Chi^2^ = 1.03, df = 1 (P = 0.31), I^2^ = 2.9%.

Across LC subtypes, the G allele variant of rs4975616 was negatively associated with the risk of NSCLC development in the overall population ([OR] = 0.85, 95%CI [0.83, 0.88]), and this negative association was present in both Caucasian populations ([OR] = 0.85, 95%CI [0.83, 0.87]) and Asian populations ([OR] = 0.90, 95%CI [0.86, 0.95]), and the strength of the negative association was higher in Caucasians than in Asians (Caucasians: [OR] = 0.85 /Asians: [OR] = 0.90, subgroup differences: P = 0.03, I^2^ = 78.4%) (Table 1, S5 Fig). In addition, the G allele variant of rs4975616 was not associated with the risk of SCLC development in the overall population ([OR] = 0.91, 95% CI [0.68, 1.23]) (Table 1, S6 Fig). The G allele variant of rs4975616 was negatively associated with the risk of developing LUAD in the overall population ([OR] = 0.86, 95%CI [0.82, 0.90]), and this negative association was present in both Caucasians ([OR] = 0.83, 95%CI [0.78, 0.88]) and Asians ([OR] = 0.92, 95%CI [0.86, 0.98]), and the strength of the negative association was stronger in Caucasians than in Asians (Caucasians: [OR] = 0.83 /Asians: [OR] = 0.92, subgroup differences: P = 0.03, I^2^ = 79.8%) (Table 1, S7 Fig). The G allele variant of rs4975616 was negatively associated with the risk of developing LUSC in the overall population ([OR] = 0.84, 95%CI [0.82, 0.87]), and this negative association was present in both Caucasians ([OR] = 0.84, 95%CI [0.82, 0.87]) and Asians ([OR] = 0.86, 95%CI [0.77, 0.96]), and the strength of this negative association was equal to that of the Asian population for the Caucasian population (Caucasians: [OR] = 0.84 /Asians: [OR] = 0.86, subgroup differences: P = 0.75, I^2^ = 0%) (Table 1, S8 Fig). Comparison of the strength of association between LUAD and LUSC showed that the G allele variant of rs4975616 was as negatively associated with the risk of developing LUAD in the overall population as it was with LUSC (LUAD: [OR] = 0.86 /LUSC: [OR] = 0.84, subgroup differences: P = 0.61, I^2^ = 0%), and also in Caucasians (LUAD: [OR] = 0.83 /LUSC: [OR] = 0.84, subgroup differences: P = 0.59, I^2^ = 0%) and Asians (LUAD: [OR] = 0.92 /LUSC: [OR] = 0.86, subgroup differences: P = 0.31, I^2^ = 2.9%) (Table 1).

### 3.4 Analysis of smoking status

**Fig 3:**
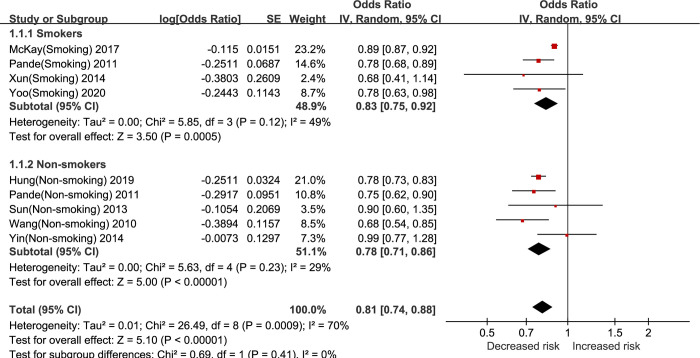
Forest plot of rs4975616 (G vs. A) for the smoking status of LC patients in the overall populations.

Subgroup analyses using smoking status of LC patients showed that in the overall population, the G allele variant of rs4975616 was negatively associated with the risk of developing LC in both smokers ([OR] = 0.83, 95%CI [0.75, 0.92]) and non-smokers ([OR] = 0.78, 95%CI [0.71, 0.86]). And the strength of this negative association did not differ between smokers and non-smokers (Smoking: [OR] = 0.83/Non-smoking: [OR] = 0.78, Subgroup differences: P = 0.41, I^2^ = 0%) (Table 2, Fig 3). In the Caucasian population, the G allele variant was negatively associated with the risk of developing LC in both smokers ([OR] = 0.85, 95% CI [0.74, 0.96]) and non-smokers ([OR] = 0.77, 95% CI [0.72, 0.81]), and the strength of this negative association did not differ between smokers and non-smokers (Smoking: [OR] = 0.85/Non-smoking:[OR] = 0.77, Subgroup differences: P = 0.18, I^2^ = 45.0%) (Table 2, S9 Fig). In the Asian population, the G allele variant was mainly negatively associated with the risk of developing LC in smokers ([OR] = 0.77, 95%CI [0.62, 0.94]) but not in non-smokers ([OR] = 0.97, 95%CI [0.78, 1.20]), and the strength of this negative association was higher in smokers than in non-smokers (Smoking: [OR] = 0.77/Non-smoking:[OR] = 0.97, Subgroup differences: P = 0.13, I^2^ = 56.9%) (Table 2, S10 Fig). Comparisons between the two populations showed that the strength of this negative association was higher in Caucasian non-smokers than in Asian non-smokers (Non-smoking Caucasians: [OR] = 0.77/Non-smoking Asians:[OR] = 0.97, Subgroup differences: P = 0.04, I^2^ = 75.3%), whereas the strength of this negative association was the same for Caucasian smokers as for Asian smokers (Smoking Caucasians: [OR] = 0.85/Smoking Asians: [OR] = 0.77, Subgroup differences: P = 0.42, I^2^ = 0%) (Table 2).

**Table 2:** Meta-analysis results of smoking status (rs4975616: Allele genetic model, G vs. A).

LC subtypes	Subgroups	Smoking status	Studies (n)	Heterogeneity test		Sample		Model	OR[95% CI]	Effect p value	Subgroup differences	
				P values	I^2^(%)	Cases (n)	Controls (n)				P values	I^2^(%)
**LC** ^a^	Overall	Smoking	4	0.12	49	50628	36770	Random	**0.83 [0.75, 0.92]**	**0.0005**	**——**	**——**
		Non-smoking	5	0.23	29	10122	16160	Random	**0.78 [0.71, 0.86]**	**<0.00001**	**——**	**——**
		Total	9	0.0009	70	60750	52930	Random	**0.81 [0.74, 0.88]**	**<0.00001**	0.41	0
	Caucasians	Smoking	2	0.05	73	48884	35382	Random	**0.85 [0.74, 0.96]**	**0.01**	**——**	**——**
		Non-smoking	3	0.49	0	8674	14712	Random	**0.77 [0.72, 0.81]**	**<0.00001**	**——**	**——**
		Total	5	**<**0.0001	83	57558	50094	Random	**0.79 [0.72, 0.87]**	**<0.00001**	0.18	45.0
	Asians	Smoking	2	0.63	0	1744	1388	Random	**0.77 [0.62, 0.94]**	**0.01**	**——**	**——**
		Non-smoking	2	0.69	0	1448	1448	Random	0.97 [0.78, 1.20]	0.75	**——**	**——**
		Total	4	0.44	0	3192	2836	Random	**0.86 [0.74, 0.99]**	**0.04**	**0.13**	**56.9**
**NSCLC** ^b^	Overall	Smoking (Asians)	2	0.67	0	1116	2412	Random	**0.75 [0.60, 0.93]**	**0.008**	**——**	**——**
		Non-smoking	3	0.06	65	1530	2554	Random	0.83 [0.62, 1.11]	0.2	**——**	**——**
		Total	5	0.19	34	2646	4966	Random	**0.79 [0.67, 0.93]**	**0.004**	0.59	0
	Caucasians	Non-smoking	1	——	——	400	1106	Random	**0.65 [0.51, 0.83]**	**0.0005**	**——**	**——**
	Asians	Smoking	2	0.67	0	1116	2412	Random	**0.75 [0.60, 0.93]**	**0.008**	**——**	**——**
		Non-smoking	2	0.67	0	1130	1448	Random	0.97 [0.77, 1.22]	0.78	**——**	**——**
		Total	4	0.39	0	2246	3860	Random	**0.84 [0.72, 0.99]**	**0.03**	**0.1**	**62.0**
**SCLC**	Overall	Smoking (Asians)	1	——	——	236	1206	Random	0.98 [0.67, 1.45]	0.94	**——**	**——**
		Non-smoking (Caucasians)	1	——	——	78	1106	Random	0.81 [0.51, 1.30]	0.39	**——**	**——**
		Total	2	0.54	0	314	2312	Random	0.91 [0.68, 1.23]	0.54	0.54	0
**LUAD** ^c^	Overall	Smoking (Asians)	1	——	——	418	1206	Random	**0.71 [0.50, 0.99]**	**0.04**	**——**	**——**
		Non-smoking	3	0.006	80	1244	2554	Random	0.78 [0.50, 1.20]	0.26	**——**	**——**
		Total	4	0.02	71	1662	3760	Random	0.76 [0.55, 1.04]	0.08	0.73	0
	Caucasians	Non-smoking	1	——	——	224	1106	Random	**0.52 [0.38, 0.71]**	**<0.0001**	**——**	**——**
	Asians	Smoking	1	——	——	418	1206	Random	**0.71 [0.50, 0.99]**	**0.04**	**——**	**——**
		Non-smoking	2	0.73	0	1020	1448	Random	0.98 [0.77, 1.23]	0.84	**——**	**——**
		Total	3	0.29	19	1438	2654	Random	0.88 [0.70, 1.09]	0.23	**0.12**	**57.8**
**LUSC** ^d^	Overall	Smoking (Asians)	1	——	——	698	1206	Random	0.78 [0.59, 1.02]	0.07	**——**	**——**
		Non-smoking	2	0.89	0	206	1506	Random	0.83 [0.58, 1.18]	0.3	**——**	**——**
		Total	3	0.95	0	904	2712	Random	**0.80 [0.64, 0.99]**	**0.04**	0.77	0
	Caucasians	Non-smoking	1	——	——	96	1106	Random	0.81 [0.53, 1.25]	0.35	**——**	**——**
	Asians	Smoking	1	——	——	698	1206	Random	0.78 [0.59, 1.02]	0.07	**——**	**——**
		Non-smoking	1	——	——	110	400	Random	0.86 [0.46, 1.61]	0.64	**——**	**——**
		Total	2	0.77	0	808	1606	Random	0.79 [0.61, 1.01]	0.06	0.77	0

LC: Lung cancer; NSCLC: Non-small-cell lung carcinoma; SCLC: Small-cell lung carcinoma; LUAD: Lung adenocarcinoma; LUSC: Lung squamous cell carcinoma.

a: LC(Smoking Caucasians) vs. LC(Smoking Asians): Test for subgroup differences: Chi^2^ = 0.65, df = 1 (P = 0.42), I^2^ = 0%; LC(Non-smoking Caucasians) vs. LC(Non-smoking Asians): Test for subgroup differences: Chi^2^ = 4.05, df = 1 **(P = 0.04), I^2^ = 75.3%**.

b: NSCLC(Non-smoking Caucasians) vs. NSCLC(Non-smoking Asians): Test for subgroup differences: Chi^2^ = 5.45, df = 1 **(P = 0.02), I^2^ = 81.7%**; NSCLC(Smoking Asians) vs. SCLC(Smoking Asians): Test for subgroup differences: Chi^2^ = 1.50, df = 1 (P = 0.22), I^2^ = 33.5%; NSCLC(Non-smoking Caucasians) vs. SCLC(Non-smoking Caucasians): Test for subgroup differences: Chi^2^ = 0.69, df = 1 (P = 0.40), I^2^ = 0%

c: LUAD(Non-smoking Caucasians) vs. LUAD(Non-smoking Asians): Test for subgroup differences: Chi^2^ = 10.04, df = 1 **(P = 0.002), I^2^ = 90.0%**; LUAD(Smoking Asians) vs. LUSC(Smoking Asians): Test for subgroup differences: Chi^2^ = 0.18, df = 1 (P = 0.67), I^2^ = 0%; LUAD(Non-smoking overall) vs. LUSC(Non-smoking overall): Test for subgroup differences: Chi^2^ = 0.05, df = 1 (P = 0.82), I^2^ = 0%; LUAD(Non-smoking Caucasians) vs. LUSC(Non-smoking Caucasians): Test for subgroup differences: Chi^2^ = 2.75, df = 1 **(P = 0.10), I^2^ = 63.6%**; LUAD(Non-smoking Asians) vs. LUSC(Non-smoking Asians): Test for subgroup differences: Chi^2^ = 0.14, df = 1 (P = 0.71), I^2^ = 0%.

d: LUSC(Non-smoking Caucasians) vs. LUSC(Non-smoking Asians): Test for subgroup differences: Chi^2^ = 0.02, df = 1 (P = 0.89), I^2^ = 0%.

Among the different LC subtypes, the G allele variant of rs4975616 was negatively associated with the risk of NSCLC incidence in both Asian smokers ([OR] = 0.75, 95%CI[0.60, 0.93]) and Caucasian non-smokers ([OR] = 0.65, 95%CI[0.51, 0.83]), whereas it was not associated with the risk of NSCLC development in Asian non-smokers ([OR] = 0.97, 95%CI[0.77, 1.22]) (Table 2, S11 and S12 Figs). Comparisons between the two populations showed that the strength of this negative association was higher in Caucasian non-smokers than in Asian non-smokers (Non-smoking Caucasians: [OR] = 0.65/Non-smoking Asians: [OR] = 0.97, Subgroup differences: p = 0.02, I^2^ = 81.7%) (Table 2). The G allele variant of rs4975616 was also negatively associated with the risk of developing LUAD in both Asian smokers ([OR] = 0.71, 95%CI[0.50, 0.99]) and Caucasian non-smokers ([OR] = 0.52, 95%CI[0.38, 0.71]), but not with the risk of developing LUAD in Asian non-smokers ([OR] = 0.98, 95% CI [0.77, 1.23]) (Table 2, S14 and S15 Figs). Comparisons between the two populations showed that the strength of this negative association was higher in Caucasian non-smokers than in Asian non-smokers (Non-smoking Caucasians: [OR] = 0.52/Non-smoking Asians: [OR] = 0.98, Subgroup differences: p = 0.002, I^2^ = 90.0%) (Table 2). In addition, the G allele variant was not negatively associated with the risk of developing SCLC in Asian smokers ([OR] = 0.98, 95%CI [0.67, 1.45]) and Caucasian non-smokers ([OR] = 0.81, 95%CI [0.51, 1.30]) (Table 2, S13 Fig). The G allele variant was not negatively associated with the risk of developing LUSC in Asian smokers ([OR] = 0.78, 95%CI [0.59, 1.02]), Asian non-smokers ([OR] = 0.86, 95%CI [0.46, 1.61]), and Caucasian non-smokers ([OR] = 0.81, 95%CI [0.53, 1.25]) (Table 2, S16 and S17 Figs).

### 3.5 Heterogeneity analysis

In analyses of LC (including subtypes), heterogeneity was predominantly present in the Caucasian population (Table 1, S5 Table) and was concentrated in the LUAD results of the Caucasian population (P = 0.005, I^2^ = 77%) (Table 1). The reasons for this may be related to factors such as different sample sizes, different countries, different study methods, different genetic testing methods, and different smoking status of the studies in these Caucasian populations. The 95% PI for each comparison was calculated using CMA v4 software, and the results of the additive, heterozygous, dominant and recessive genetic models and the results of the LUAD for the Caucasian population were found to be changed (95% PI included 1) (Table 1, S5 Table and S18 and S19 Figs), which suggests that these results can be somewhat unstable due to the presence of heterogeneity. In addition, the LUAD result of Asian populations also showed such change (95% PI included 1) (Table 1), the reason of which may be related to the small sample size.

In the subgroup analyses of the patients’ smoking status, heterogeneity was still predominantly present in the Caucasian population (I^2^>50%) (Table 2). The calculation of 95% PI showed changes in the LC results for smokers and non-smokers (95% PI included 1) (S6 Table and S19 Fig), and the reasons for these changes may be related to the small number of studies, small sample sizes, and a certain degree of heterogeneity. In addition, the remaining subgroups could not have their 95% PI calculated accurately due to the small number of included studies.

Meta-regression using age and sex ratio (male%) of LC patients and minor allele frequency (MAF) of controls as independent variables revealed that none of these factors was the main source of heterogeneity (P>0.05) (S20 Fig). Therefore, the generation of heterogeneity may still be related to those factors mentioned before.

### 3.6 Publication bias

Most of the funnel plots appear to be symmetrical (S21 and S22 Figs). In LC, although the Egger’s test results of G vs. A(Overall), GA vs. AA(Overall and Caucasians) and GG+GA vs. AA(Overall) were all less than 0.05 (P_Egger_<0.05), their Begg’s test results were all more than 0.05 (P_Begg_>0.05). The Begg’s test result of GG vs. GA+AA(Asians) was less than 0.05 (P_Begg_<0.05), but its Egger’s test result was more than 0.05 (P_Egger_>0.05) (Table 1, S5 Table). In LUAD, although the Egger’s test results for G vs. A (Overall and Caucasians) were all less than 0.05 (P_Egger_<0.05), their Begg’s test results were all more than 0.05 (P_Begg_>0.05) (Table 1). From these results, it cannot be suggested that they are biased. In addition, the results of all other models were not significantly biased (P_Egger_<0.05 and P_Egger_<0.05) (Table 1, S5 and S6 Tables).

### 3.7 Sensitivity analysis

The results of sensitivity analysis of all genetic models showed no significant sensitivity in any of the studies, indicating that there was no significant difference in the result of the meta-analysis after removing any study (S7 Table and S23 and S24 Figs).

### 3.8 Trial sequential analysis (TSA)

The TSA analysis showed that the Z-curve (blue line) crossed both the traditional boundary (green dashed line) and the TSA boundary (red line), proving that the results of LC, NSCLC, LUAD, LUSC and LC smoking subgroups were stable and credible (S25 and S26 Figs).

## 4. Discussion

In a previous large GWAS, rs4975616 (A >G) located in the TERT-CLPTM1L region had been found to be associated with the risk of LC [25]. However, several other studies had shown inconsistent results [30–34]. Therefore, the association of the variant of rs4975616 with the risk of LC currently lacked a unifying conclusion. The aim of this meta-analysis was to clarify its association with LC and the differences in this association between patients of different ethnicities (Caucasian populations and Asian populations), different subtypes of LC, and different smoking status. The results showed the negative association between the G allele variant of rs4975616 and the risk of developing LC, and this negative association was present in both Caucasian and Asian populations. Previous studies in Caucasian [11, 25–27, 37] and Asian populations [28, 29, 40] reported similar results. These results confirm that the minor G allele variant of rs4975616 reduces the risk of LC in both populations, while its major A allele (risk allele) variant increases the risk of LC in both populations. Comparing the two populations, we found that the G allele variant of rs4975616 was more strongly negatively associated with the risk of LC development in the Caucasian population than in the Asian population, suggesting that the variant of rs4975616 was differentially associated with the strength of the risk of LC development in the two populations, and that Caucasians were more likely to have a higher risk of LC development due to the variant of rs4975616. In addition, variants in rs4975616 did not seem to be negatively associated with the risk of LC development in Asian populations in any of the additive, heterozygous, dominant, and recessive models, implying that variants in rs4975616 may not be associated with the risk of LC development in Asian populations. However, it is worth noting that some studies did not have complete genotypes reported, resulting in a smaller number of Asian populations included in these models (n = 4) and a smaller sample size of patients. Therefore, this result is inconclusive and needs to be verified by further inclusion of more studies.

Across different LC subtypes, our results demonstrated negative associations of the G allele variant of rs4975616 with the risk of developing NSCLC, LUAD, and LUSC, and these negative associations were all present in both Caucasian and Asian populations. These results were the same as those of previous studies in Caucasian populations [11, 38, 39, 40] and Asian populations [40]. This evidence confirms that both populations are at risk for developing NSCLC, LUAD, and LUSC due to variants in rs4975616. Comparing the two populations, we found that the strength of the negative association between the G allele variant of rs4975616 and the risk of developing NSCLC and LUAD was higher in Caucasian than in Asian populations, indicating that the strength of the association between the variant of rs4975616 and the risk of developing NSCLC and LUAD differed between the two populations, and that Caucasian populations had a higher risk of developing NSCLC and LUAD due to the variant of rs4975616. In addition, the strength of the negative association of LUSC incidence risk in the Caucasian population was the same as that in the Asian population, suggesting that the risk of LUSC incidence is high in both populations. Comparisons between LUAD and LUSC revealed the same strength of negative association between the G allele variant of rs4975616 and the risk of developing LUAD as that of LUSC, and in both populations. These results were broadly consistent with those of previous studies [11, 39, 40]. It suggests that both subtypes of NSCLC are at high risk of developing in both Caucasian and Asian populations, and that both Caucasian and Asian populations are likely to be at risk of developing LUAD or LUSC due to the rs4975616 variant. In addition, our results found that the G allele variant of rs4975616 did not reduce the risk of SCLC, suggesting that the rs4975616 variant may not be associated with the risk of SCLC.

The results of epidemiologic investigations had shown that although smoking was identified as a major environmental risk factor for LC worldwide, only a small percentage of smokers developed LC during their lifetime. In contrast, a large percentage of LC cases had no history of smoking [41, 42]. LC among never-smokers differed from LC among smokers in that a large proportion of LC patients among never-smokers carried genetic variants of oncogenes [43]. Previous studies had shown that genetic susceptibility to LC in never-smokers was associated with genetic variants with pan-cancer risk implications, and that gene-environment interactions were important in LC etiology [37]. In order to clarify whether smoking or non-smoking can cause the CLPTM1L rs4975616 variant and thus the risk of developing LC, we conducted a subgroup analysis of patients’ smoking status. We found that the G allele variant of rs4975616 was negatively associated with the risk of developing LC in both smokers and non-smokers, and the strength of this negative association did not differ between smokers and non-smokers. This confirms that smoking is indeed an important risk factor for the rs4975616 variant leading to LC, but it is not the only factor, because other factors like non-smoking can also cause the rs4975616 variant leading to LC. Previous evidence suggested that patient’s education, BMI, previous diagnosis of COPD, occupational exposure to pesticides, duration of smoking, exposure to high levels of cooking emissions, and dietary factors (including less fish and shrimp, vegetables, soy products, and nuts), as well as a high consumption of meat, were all associated with the development of LC [44]. Thus, LC is a multi-causal disease triggered by a combination of smoking, genetic, environmental and lifestyle factors.

In each ethnic group, we found this negative association in both Caucasian smokers and Caucasian non-smokers, and there was no difference in the strength of this negative association between them. This was the same as previous findings in Caucasian smokers [26] and Caucasian non-smokers [37, 38]. It implies that regardless of whether the Caucasian population smokes or not, they are likely to be at risk for LC due to the variant in rs4975616. Therefore, smoking remains an important risk factor for causing the rs4975616 variant and lead to LC in the Caucasian population, yet other factors like non-smoking can also induce the rs4975616 variant and lead to LC in Caucasians. However, in Asian populations, we found that this negative association appeared to be present only in smokers and not in non-smokers, and the strength of this negative association was higher in smokers than in non-smokers. Previous studies in Asian smokers [29] and Asian non-smokers [34] reported similar results. These results suggest that smoking is also a major risk factor for causing variants in rs4975616 and lead to LC in Asian populations, and other factors like non-smoking do not seem to contribute to the development of LC. However, it is worth noting that the number of Asian non-smokers included in this study was small (n = 2), so this conclusion needs to be verified by including more studies. In addition, our results also showed that the strength of the negative association for the risk of LC development was higher in Caucasian non-smokers than in Asian non-smokers, suggesting that the Caucasian population is more at risk of LC development due to other factors like non-smoking that cause variants in rs4975616 and lead to LC, and also provides further evidence that other factors like non-smoking that cause variants in rs4975616 and lead to LC development in Asian populations are at a lower risk.

Among different LC subtypes, we found that the G allele variant of rs4975616 was negatively associated with the risk of developing NSCLC (LUAD) mainly in Caucasian non-smokers and Asian smokers, suggesting that this negative risk association caused by the G allele variant in Caucasian non-smokers and Asian smokers is mainly concentrated in the LC subtype of NSCLC (LUAD). Previous studies have reported similar results [29, 38]. Therefore, in the Caucasian population, other factors like non-smoking can cause the rs4975616 variant and lead to NSCLC (LUAD), whereas smoking is the major risk factor for causing rs4975616 variation and thus NSCLC(LUAD) in Asian populations. In addition, we did not find the negative association between the G allele variant of rs4975616 and the risk of developing NSCLC (LUAD) in Asian non-smokers, the result that was the same as that of some previous studies [32, 34]. However, the evidence [45] had already confirmed that the common genetic variation of TERT-CLPTM1L was associated with the risk of LUAD in non-smoking Asian women, and the number of Asian non-smokers included in our study was small (n = 2). Therefore, this result is inconclusive and more studies need to be included for verification. There is also the fact that we did not look for data results on Caucasian smokers, and therefore could not determine the association between the rs4975616 variant and the risk of NSCLC (LUAD) development in Caucasian smokers.

In addition, we found no risk-negative association between the G allele variant of rs4975616 and the development of SCLC and LUSC in both smokers and non-smokers. However, because of the small number of included studies for both subtypes of LC, the association between the G allele variant and SCLC and LUSC needs to be verified by further studies.

Limitations: ①The meta-analysis was based on the results of studies of different ethnicities, different LC subtypes and different smoking status, so some heterogeneity and publication bias will inevitably exist; ②The genetic testing and genotyping methods used in all the studies were different, and some heterogeneity and publication bias will also exist; ③In terms of the sample size, overall the present study was still sufficient. However, after subgroup analysis based on different LC subtypes and different ethnicities, the sample size was still on the low side, especially for LUAD and SCLC. This will inevitably produce some false-negative results for the results of LUAD and SCLC; ④Although the current study discussed in detail about the effects of smoking, genes, environment, lifestyle and other factors on LC, the sample size included in the subgroup analysis of smoking status was still small. Therefore, the reliability of the results of the association between smoking or non-smoking and the risk of LC (including each LC subtype) may be affected to a certain extent; ⑤Because fewer results have been reported for other ethnicities (African population, mixed population), the results of the data from these populations were not analyzed in the current study, and therefore these results do not represent the association of the risk of LC among all ethnicities; ⑥All the literature included in this study was in English and no literature in other languages was included.

## 5. Conclusion

The G allele variant of rs4975616 is negatively associated with the risk of LC and NSCLC (LUAD, LUSC), and it is more strongly associated with the risk of LC and NSCLC (LUAD) in Caucasians than in Asians. Therefore, compared with Asian populations, Caucasians are more likely to have a higher risk of developing LC and NSCLC (LUAD) due to the rs4975616 variant. In Caucasian populations, smoking and other factors like non-smoking contribute to rs4975616 variations leading to LC, and other factors like non-smoking also induce rs4975616 variations leading to NSCLC (LUAD). In Asian populations, smoking is the major risk factor for the induction of rs4975616 variations leading to LC and NSCLC(LUAD). Therefore, the risk factors for the development of LC are different between these two populations.

## Supporting information

S1 Fig Forest plot of rs4975616 (GG vs. AA) for LC. (DOCX)

S2 Fig Forest plot of rs4975616 (GA vs. AA) for LC. (DOCX)

S3 Fig Forest plot of rs4975616 (GG+GA vs. AA) for LC. (DOCX)

S4 Fig Forest plot of rs4975616 (GG vs. GA+AA) for LC. (DOCX)

S5 Fig Forest plot of rs4975616 (G vs. A) for NSCLC. (DOCX)

S6 Fig Forest plot of rs4975616 (G vs. A) for SCLC. (DOCX)

S7 Fig Forest plot of rs4975616 (G vs. A) for LUAD. (DOCX)

S8 Fig Forest plot of rs4975616 (G vs. A) for LUSC. (DOCX)

S9 Fig Forest plot of rs4975616 (G vs. A) for the smoking status of LC patients in Caucasians. (DOCX)

S10 Fig Forest plot of rs4975616 (G vs. A) for the smoking status of LC patients in Asians. (DOCX)

S11 Fig Forest plot of rs4975616 (G vs. A) for the smoking status of NSCLC patients in the overall populations. (DOCX)

S12 Fig Forest plot of rs4975616 (G vs. A) for the smoking status of NSCLC patients in Caucasians or Asians. (DOCX)

S13 Fig Forest plot of rs4975616 (G vs. A) for the smoking status of SCLC patients in overall populations. (DOCX)

S14 Fig Forest plot of rs4975616 (G vs. A) for the smoking status of LUAD patients in the overall populations. (DOCX)

S15 Fig Forest plot of rs4975616 (G vs. A) for the smoking status of LUAD patients in Caucasians or Asians. (DOCX)

S16 Fig Forest plot of rs4975616 (G vs. A) for the smoking status of LUSC patients in the overall populations. (DOCX)

S17 Fig Forest plot of rs4975616 (G vs. A) for the smoking status of LUSC patients in Caucasians or Asians. (DOCX)

S18 Fig The 95% prediction interval for the association of rs4975616 with LC. A: G vs. A; B: GG vs. AA; C: GA vs. AA; D: GG+GA vs. AA; E: GG vs. GA+AA.
(DOCX)

S19 Fig The 95% prediction interval for the association of rs4975616(G vs. A) with LC of different ethnicity/pathological subtypes/smoking status. A: NSCLC; B: SCLC; C: LUAD; D: LUSC; E: LC Smokers; F: LC Non-smokers.
(DOCX)

S20 Fig The results of meta-regression. A: age of LC patients, set: age ≥ 60 or < 60; B: sex ratio (male%) of LC patients, set: male% ≥ 60% or < 60%; C: minor allele frequency (MAF) of controls, set: MAF ≥ 0.4 or <0.4.
(DOCX)

S21 Fig Publication bias for the association of rs4975616 with LC. A: G vs. A; B: GG vs. AA; C: GA vs. AA; D: GG+GA vs. AA; E: GG vs. GA+AA.
(DOCX)

S22 Fig Publication bias for the association of rs4975616(G vs. A) with LC of different ethnicity/pathological subtypes/smoking status. A: NSCLC; B: LUAD; C: LUSC; D: LC Smoking status.
(DOCX)

S23 Fig Results of sensitivity analysis of rs4975616 in association with LC. A: G vs. A; B: GG vs. AA; C: GA vs. AA; D: GG+GA vs. AA; E: GG vs. GA+AA.
(DOCX)

S24 Fig Results of sensitivity analysis of rs4975616(G vs. A) in association with LC of different ethnicity/pathological subtypes/smoking status. A: NSCLC; B: LUAD; C: LUSC; D: LC Smoking status.
(DOCX)

S25 Fig TSA results for the association of rs4975616 with LC. A: G vs. A; B: GG vs. AA; C: GA vs. AA; D: GG+GA vs. AA; E: GG vs. GA+AA.
(DOCX)

S26 Fig TSA results for the association of rs4975616(G vs. A) with LC in different pathological subtypes/smoking status. A: NSCLC; B: LUAD; C: LUSC; D: LC Smokers; E: LC Non-smokers.
(DOCX)

S1 Table Literature search strategy and search results. A: PubMed search strategy. B: A numbered table of all studies identified in the literature search.
(DOCX)

S2 Table Basic features of the included study (1). (DOCX)

S3 Table Basic features of the included study (2). (DOCX)

S4 Table Newcastle Ottawa scale (NOS). (DOCX)

S5 Table The LC results of additive, heterozygous, dominant and recessive genetic models in rs4975616. (DOCX)

S6 Table The publication bias and 95% prediction interval for the association of rs4975616(G vs. A) with LC of different smoking status. (DOCX)

S7 Table The results of sensitivity analysis. (DOCX)

## Abbreviations

LC: Lung cancer
GWAS: Genome-wide association studies
CLPTM1L: Cleft lip and cleft palate transmembrane protein 1
TERT: Telomerase reverse transcriptase
OR: Odds ratio
95% CI: 95% Confidence interval
HWE: Hardy-Weinberg equilibrium
NOS: Newcastle Ottawa scale
TSA: Trial sequential analysis
NSCLC: Non-small-cell lung carcinoma
LUAD: Lung adenocarcinoma
LUSC: Lung squamous cell carcinoma
SCLC: Small cell lung carcinoma
