# Supplementary figures and images for: Differences in the risk association of TERT-CLPTM1L rs4975616 (A>G) with lung cancer between Caucasian and Asian populations: A meta-analysis
Source: PLoS One. 2024 Sep 10;19(9):e0309747. doi: 10.1371/journal.pone.0309747 (PMC11386447; doi:10.1371/journal.pone.0309747)

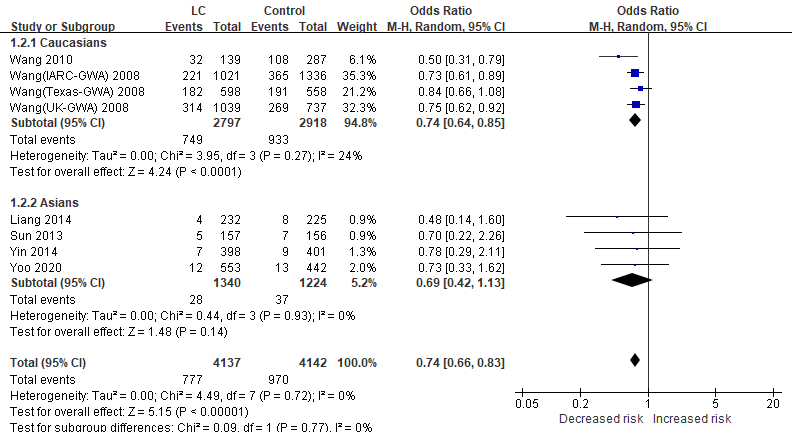


**S1 Fig. Forest plot of rs4975616 (GG vs.AA ) for LC.**

Supplement: S1 Fig — (DOCX) [file pone.0309747.s001.docx]

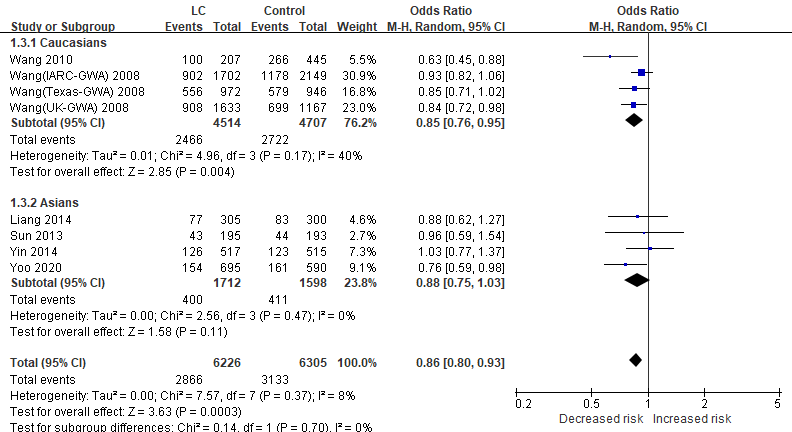


**S2 Fig. Forest plot of rs4975616 (GA vs.AA ) for LC.**

Supplement: S2 Fig — (DOCX) [file pone.0309747.s002.docx]

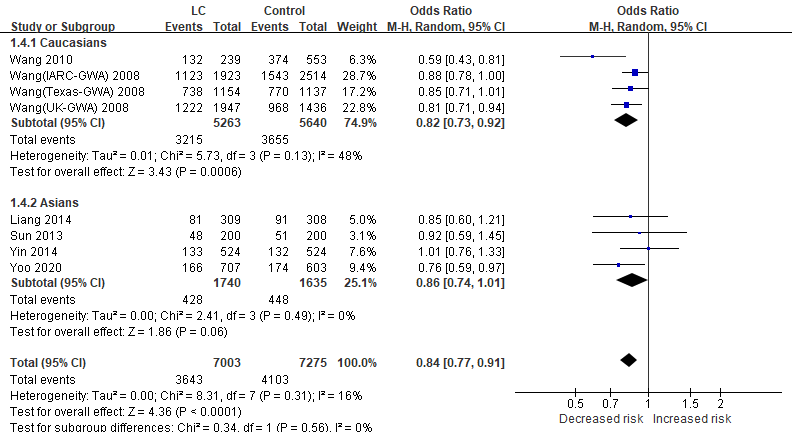


**S3 Fig. Forest plot of rs4975616 (GG+GA vs.AA ) for LC.**

Supplement: S3 Fig — (DOCX) [file pone.0309747.s003.docx]

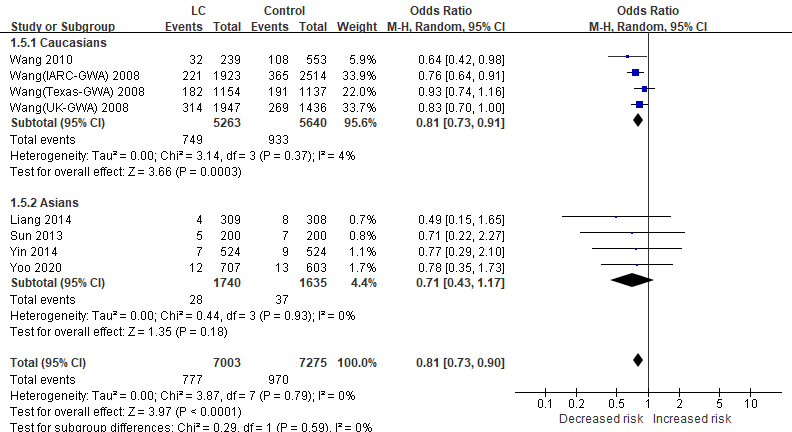


**S4 Fig. Forest plot of rs4975616 (GG vs.GA+AA ) for LC.**

Supplement: S4 Fig — (DOCX) [file pone.0309747.s004.docx]

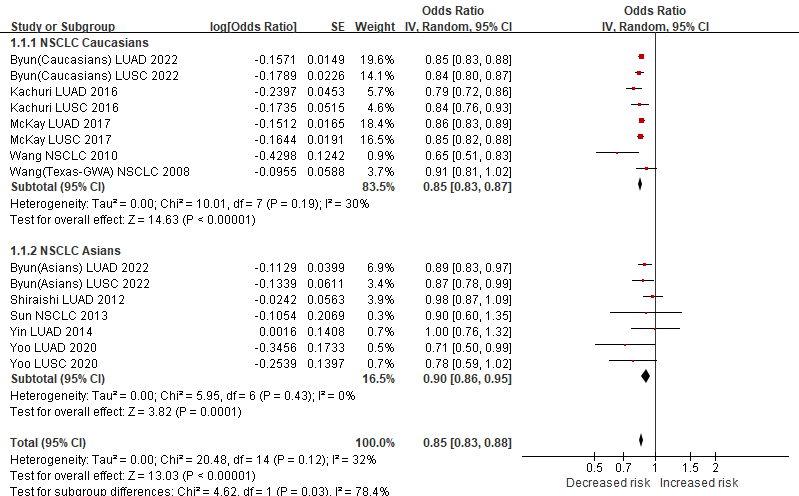


**S5 Fig. Forest plot of rs4975616 (G vs.A ) for NSCLC.**

Supplement: S5 Fig — (DOCX) [file pone.0309747.s005.docx]

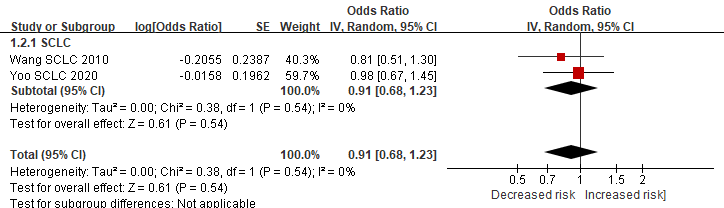


**S6 Fig. Forest plot of rs4975616 (G vs.A ) for SCLC.**

Supplement: S6 Fig — (DOCX) [file pone.0309747.s006.docx]

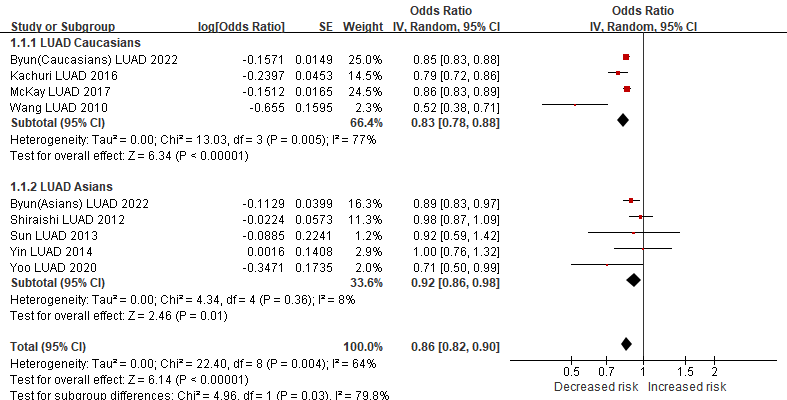


**S7 Fig. Forest plot of rs4975616 (G vs.A ) for LUAD.**

Supplement: S7 Fig — (DOCX) [file pone.0309747.s007.docx]

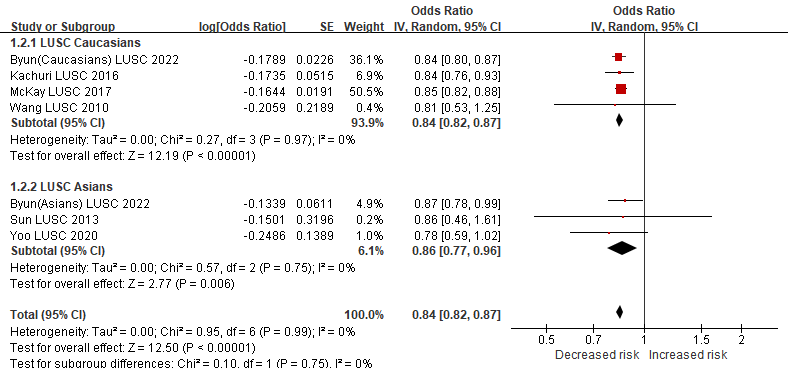


**S8 Fig. Forest plot of rs4975616 (G vs.A ) for LUSC.**

Supplement: S8 Fig — (DOCX) [file pone.0309747.s008.docx]

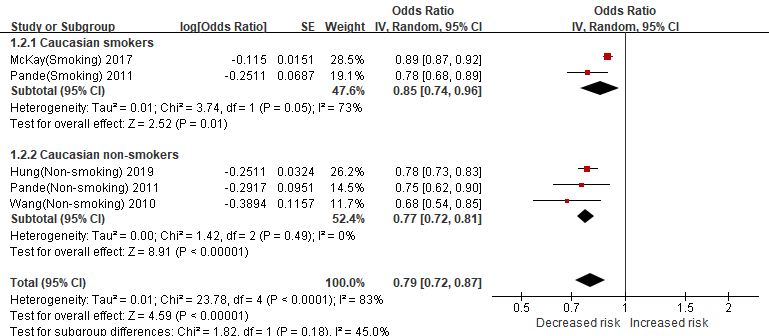


**S9 Fig. Forest plot of rs4975616 (G vs.A ) for the smoking status of LC patients in Caucasians.**

Supplement: S9 Fig — (DOCX) [file pone.0309747.s009.docx]

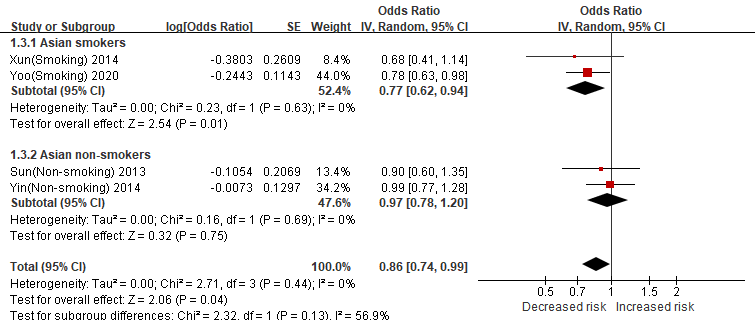


**S10 Fig. Forest plot of rs4975616 (G vs.A ) for the smoking status of LC patients in Asians.**

Supplement: S10 Fig — (DOCX) [file pone.0309747.s010.docx]

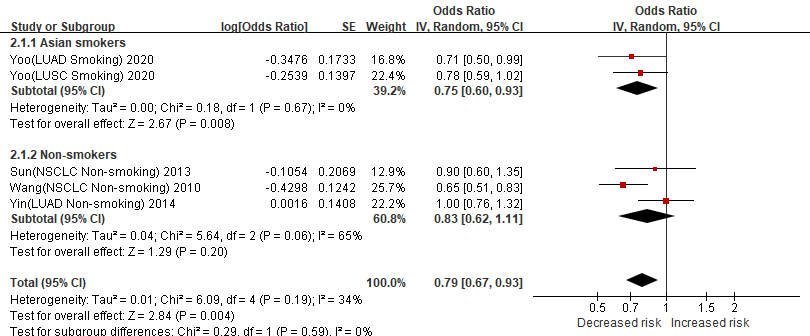


**S11 Fig. Forest plot of rs4975616 (G vs.A ) for the smoking status of NSCLC patients in the overall populations.**

Supplement: S11 Fig — (DOCX) [file pone.0309747.s011.docx]

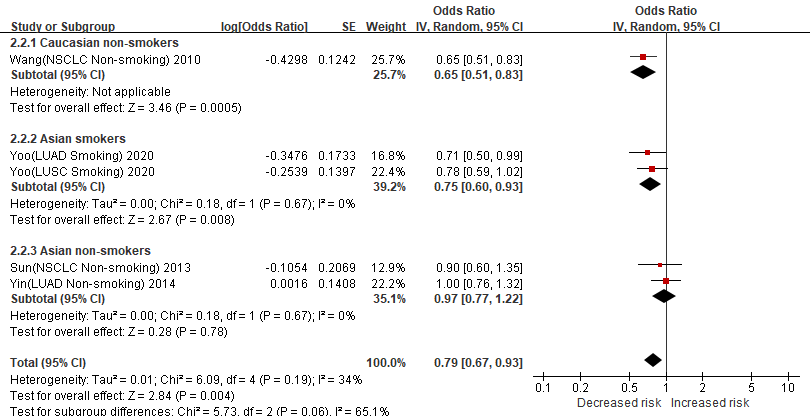


**S12 Fig. Forest plot of rs4975616 (G vs.A ) for the smoking status of NSCLC patients in Caucasians or Asians.**

Supplement: S12 Fig — (DOCX) [file pone.0309747.s012.docx]

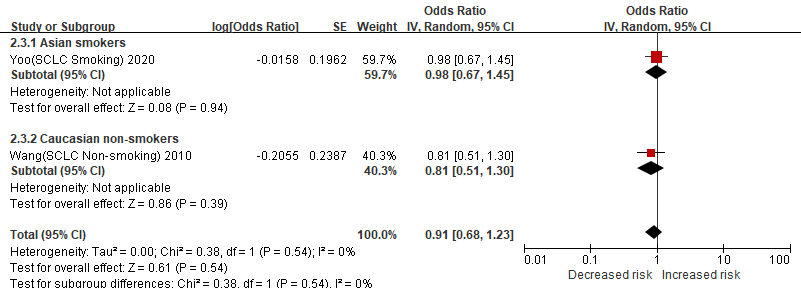


**S13 Fig. Forest plot of rs4975616 (G vs.A ) for the smoking status of SCLC patients in overall populations.**

Supplement: S13 Fig — (DOCX) [file pone.0309747.s013.docx]

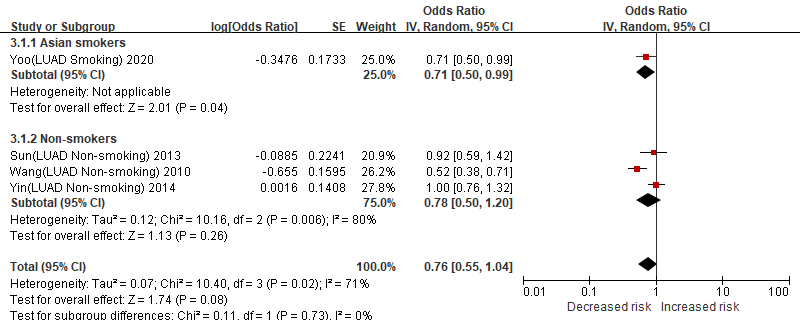


**S14 Fig. Forest plot of rs4975616 (G vs.A ) for the smoking status of LUAD patients in the overall populations.**

Supplement: S14 Fig — (DOCX) [file pone.0309747.s014.docx]

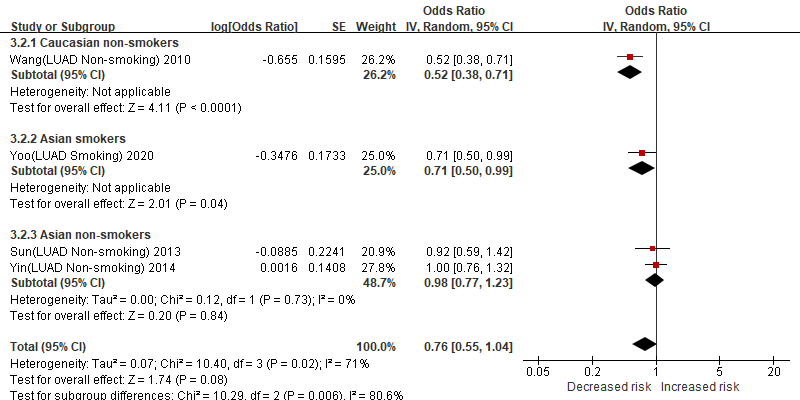


**S15 Fig. Forest plot of rs4975616 (G vs.A ) for the smoking status of LUAD patients in Caucasians or Asians.**

Supplement: S15 Fig — (DOCX) [file pone.0309747.s015.docx]

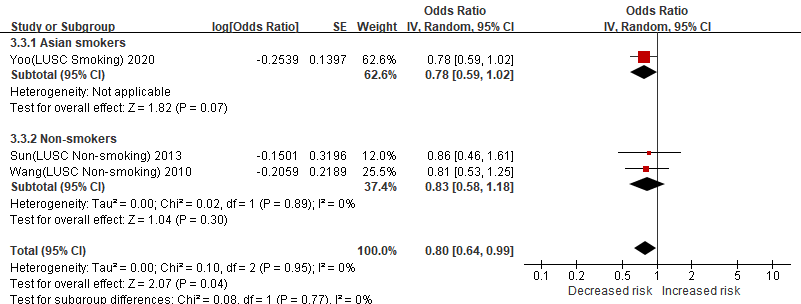


**S16 Fig. Forest plot of rs4975616 (G vs.A ) for the smoking status of LUSC patients in the overall populations.**

Supplement: S16 Fig — (DOCX) [file pone.0309747.s016.docx]

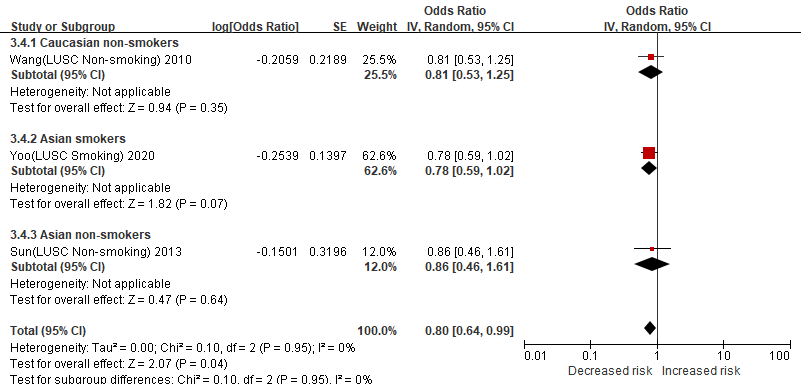


**S17 Fig. Forest plot of rs4975616 (G vs.A ) for the smoking status of LUSC patients in Caucasians or Asians.**

Supplement: S17 Fig — (DOCX) [file pone.0309747.s017.docx]
