# Supplementary material for: Differences in the risk association of TERT-CLPTM1L rs4975616 (A>G) with lung cancer between Caucasian and Asian populations: A meta-analysis
Source: PLoS One. 2024 Sep 10;19(9):e0309747. doi: 10.1371/journal.pone.0309747 (PMC11386447; doi:10.1371/journal.pone.0309747)
Supplement: S19 Fig — A: NSCLC; B: SCLC; C: LUAD; D: LUSC; E: LC Smokers; F: LC Non-smokers. (DOCX) [file pone.0309747.s019.docx]

| A | 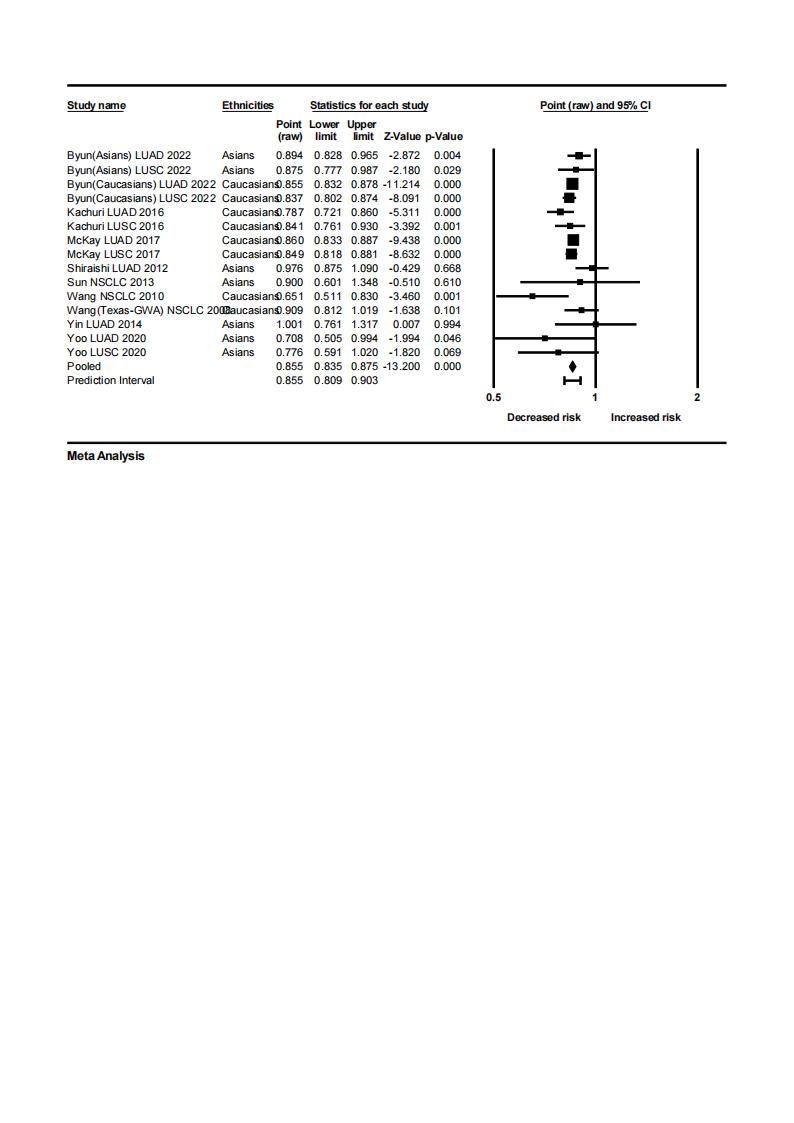 |
| --- | --- |
| B | 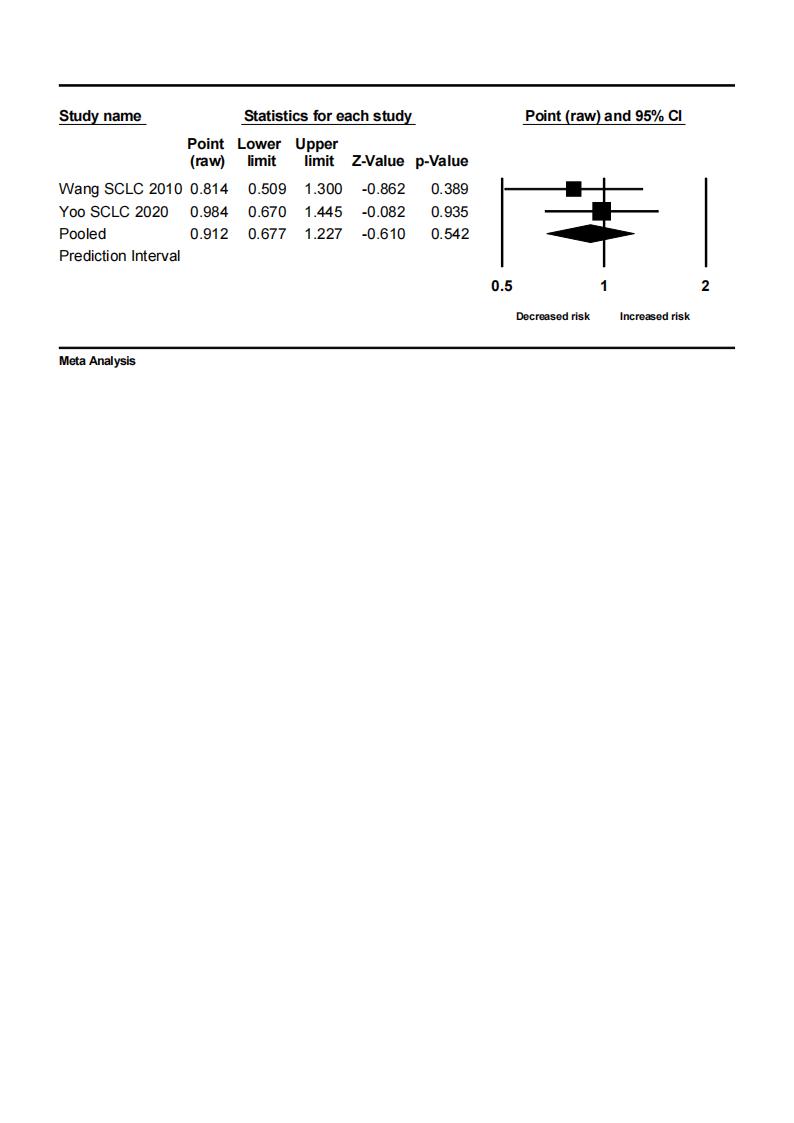 |
| C | 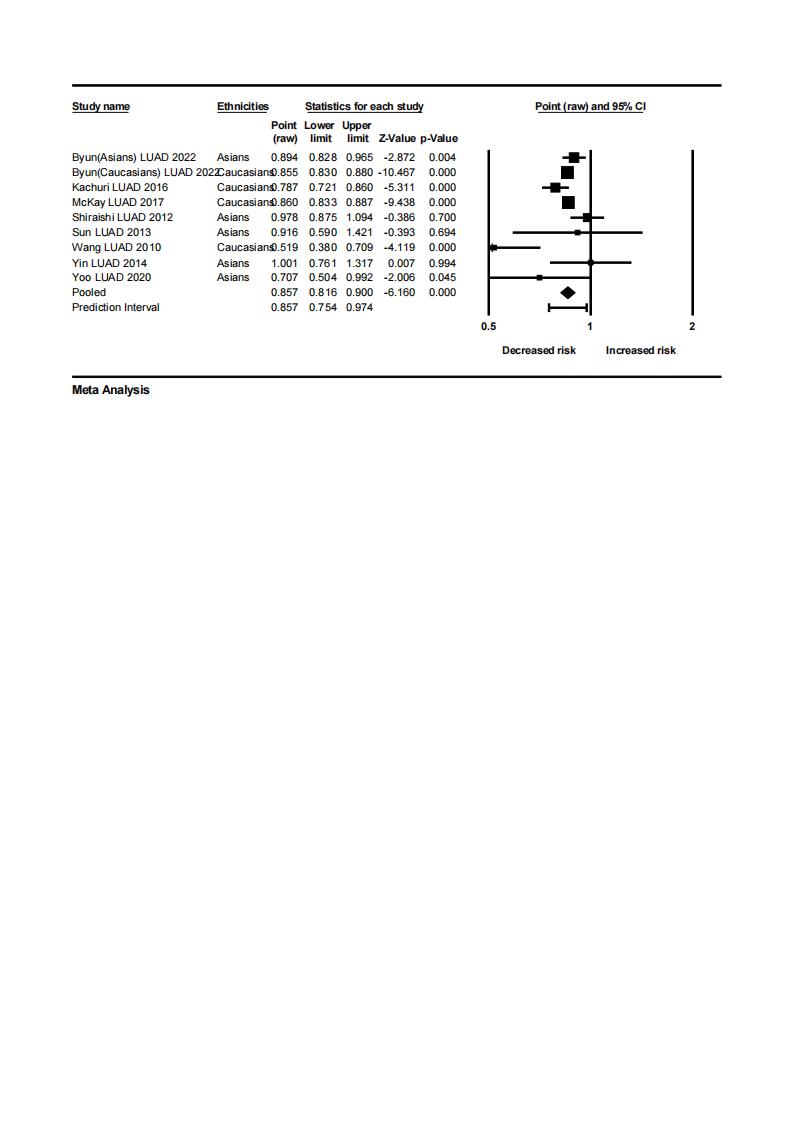 |
| D | 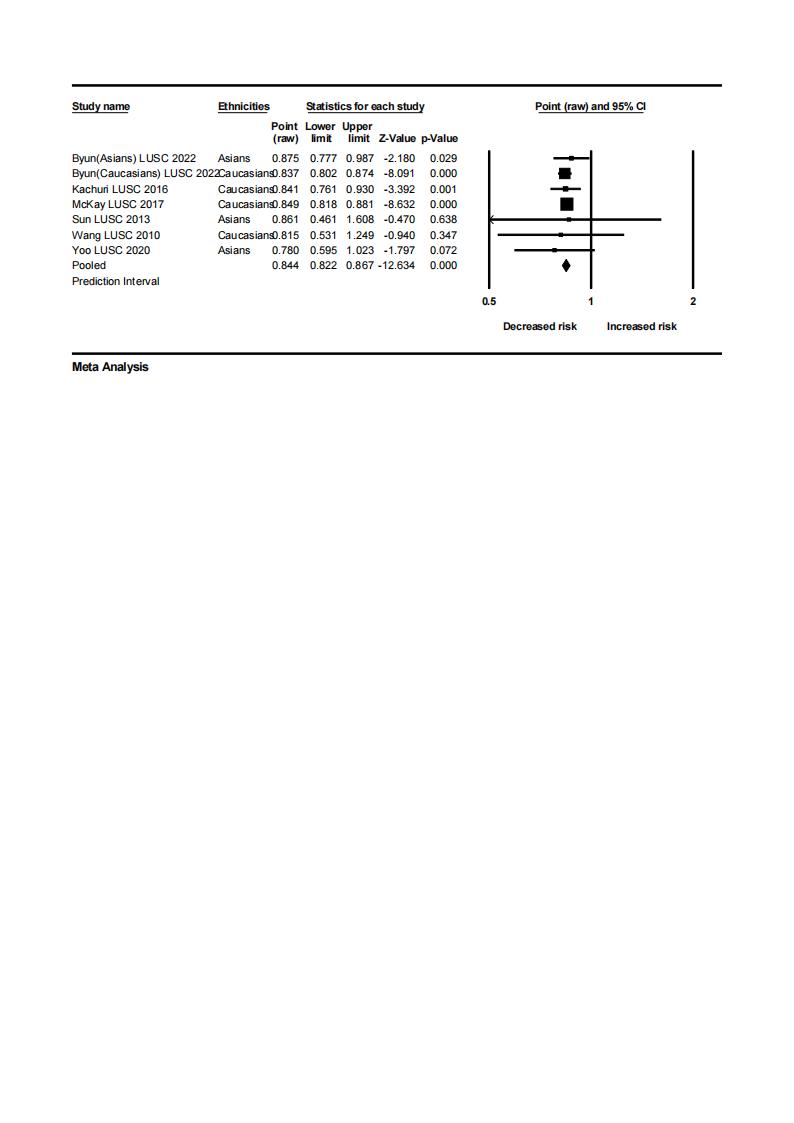 |
| E | 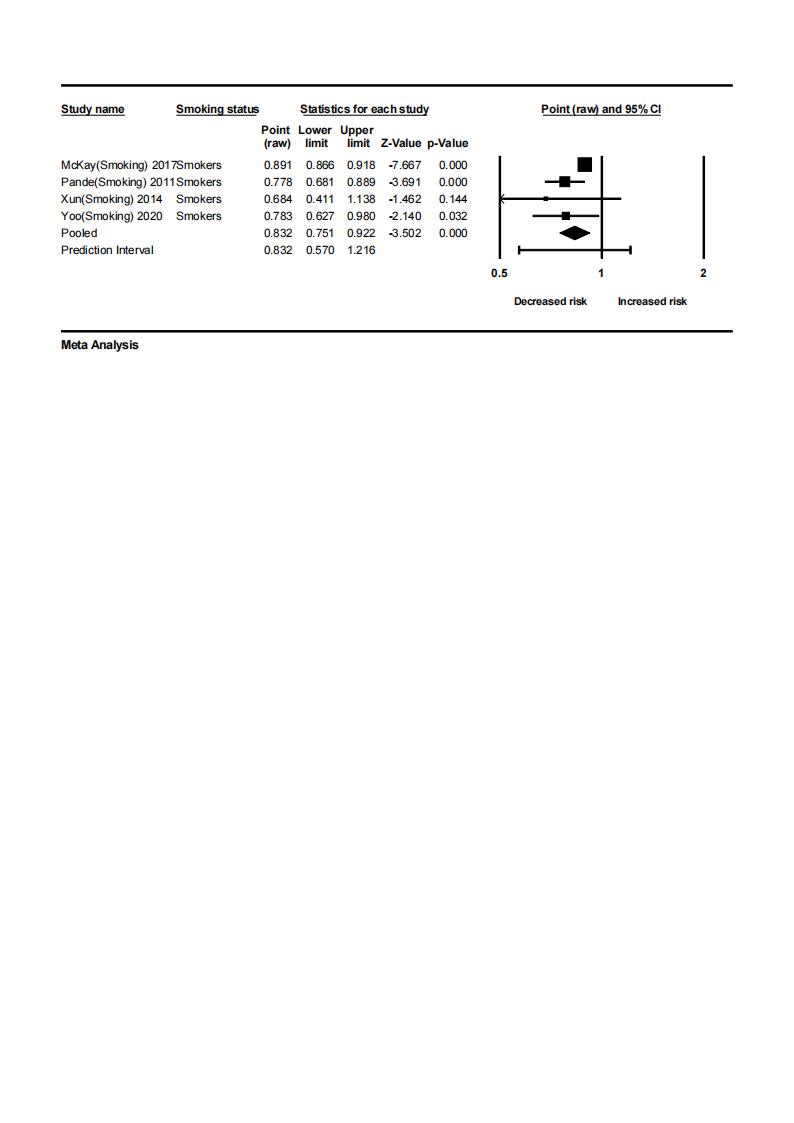 |
| F | 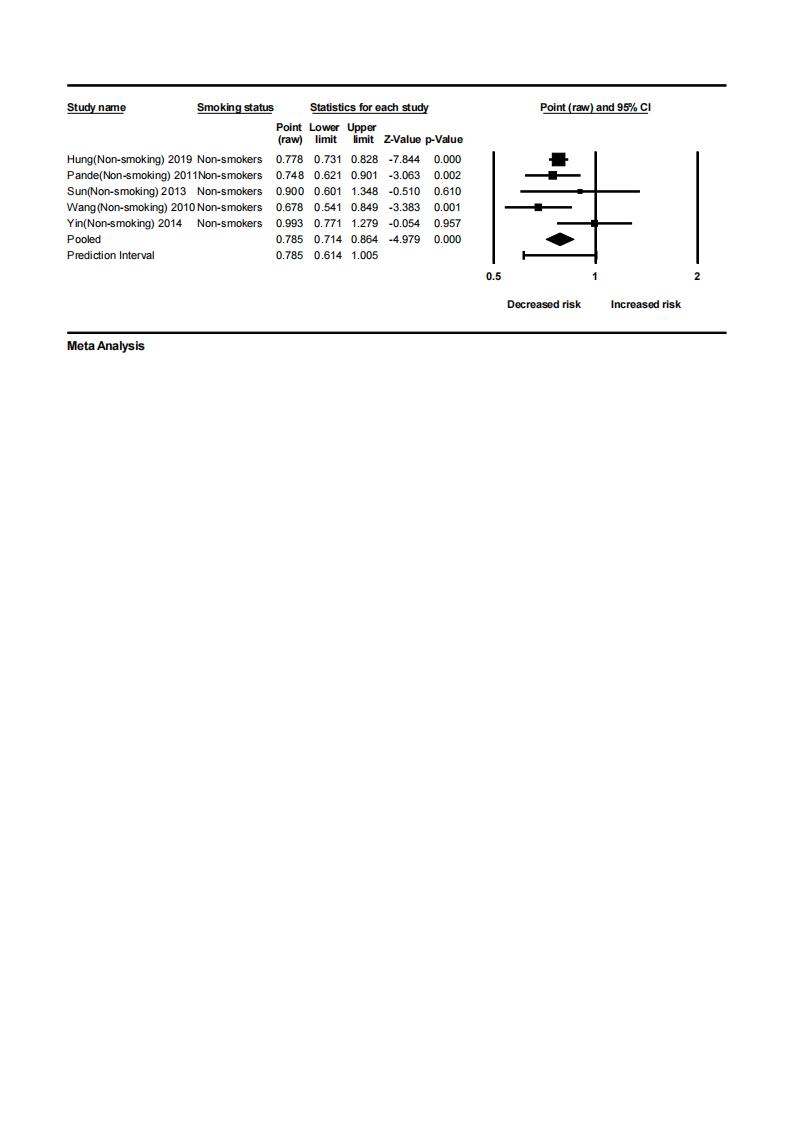 |

**S19 Fig. The 95% Prediction Interval for the association of rs4975616(G vs.A) with LC of different ethnicity/pathological subtypes/smoking status.**

A:NSCLC; B:SCLC; C:LUAD; D:LUSC; E:LC Smokers; F:LC Non-smokers.
