# Supplementary material for: Differences in the risk association of TERT-CLPTM1L rs4975616 (A>G) with lung cancer between Caucasian and Asian populations: A meta-analysis
Source: PLoS One. 2024 Sep 10;19(9):e0309747. doi: 10.1371/journal.pone.0309747 (PMC11386447; doi:10.1371/journal.pone.0309747)
Supplement: S20 Fig — A: age of LC patients, set: age ≥ 60 or < 60; B: sex ratio (male%) of LC patients, set: male% ≥ 60% or < 60%; C: minor allele frequency (MAF) of controls, set: MAF ≥ 0.4 or <0.4. (DOCX) [file pone.0309747.s020.docx]

| A |  |  |
| --- | --- | --- |
| B |  |  |
| C |  |  |

**S20 Fig. The results of Meta-regression.**

A: age of LC patients, set: age ≥ 60 or < 60; B: sex ratio (male%) of LC patients, set: male% ≥ 60% or < 60%; C: minor allele frequency (MAF) of controls, set: MAF ≥ 0.4 or <0.4.
