# Supplementary material for: Differences in the risk association of TERT-CLPTM1L rs4975616 (A>G) with lung cancer between Caucasian and Asian populations: A meta-analysis
Source: PLoS One. 2024 Sep 10;19(9):e0309747. doi: 10.1371/journal.pone.0309747 (PMC11386447; doi:10.1371/journal.pone.0309747)
Supplement: S21 Fig — A: G vs. A; B: GG vs. AA; C: GA vs. AA; D: GG+GA vs. AA; E: GG vs. GA+AA. (DOCX) [file pone.0309747.s021.docx]

| A | 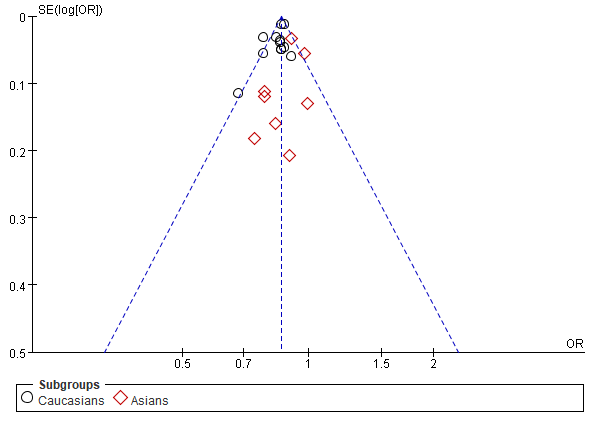 |  |  |
| --- | --- | --- | --- |
| B | **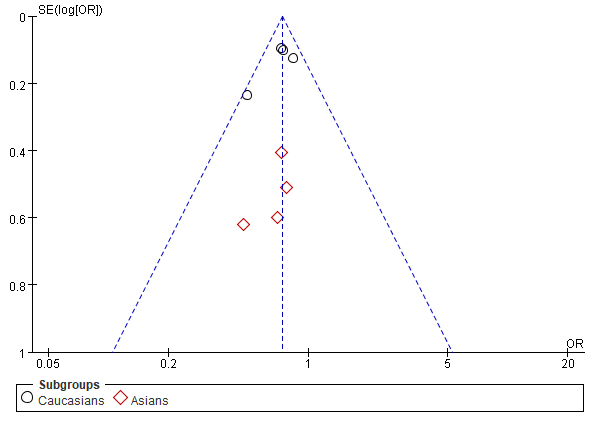** |  |  |
| C | **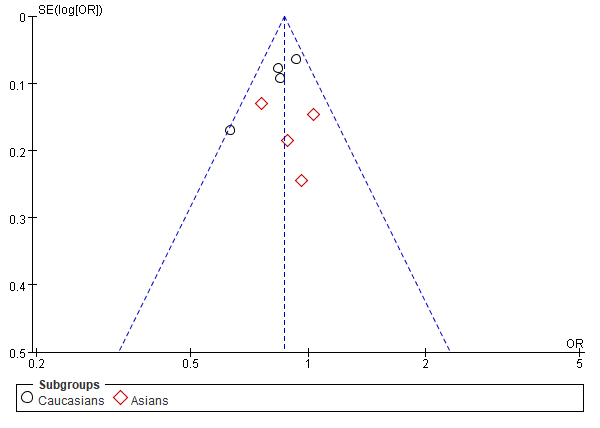** |  |  |
| D | **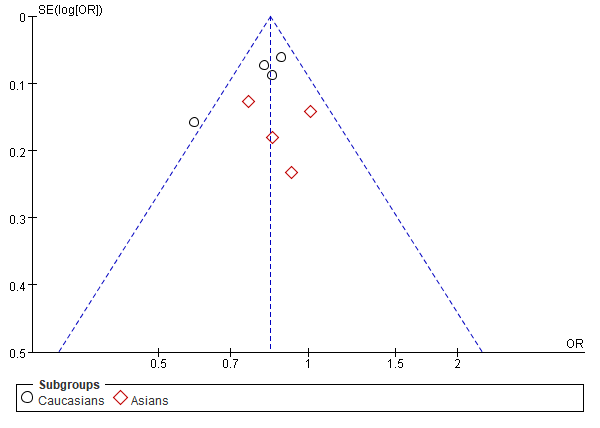** |  |  |
| E | **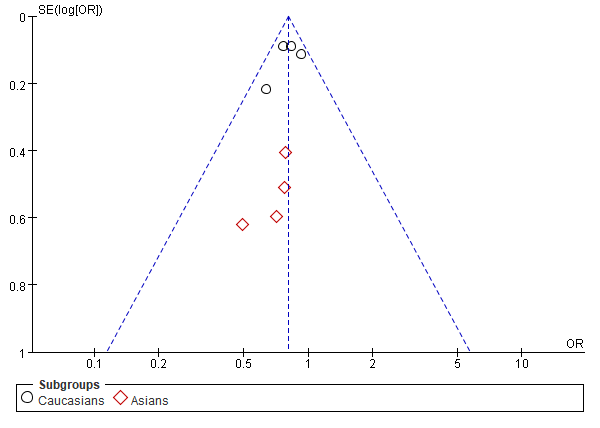** |  |  |

**S21 Fig. Publication bias for the association of rs4975616 with LC.**

A:G vs.A; B:GG vs.AA; C:GA vs.AA; D:GG+GA vs.AA; E:GG vs.GA+AA.
