# Supplementary material for: Differences in the risk association of TERT-CLPTM1L rs4975616 (A>G) with lung cancer between Caucasian and Asian populations: A meta-analysis
Source: PLoS One. 2024 Sep 10;19(9):e0309747. doi: 10.1371/journal.pone.0309747 (PMC11386447; doi:10.1371/journal.pone.0309747)
Supplement: S23 Fig — A: G vs. A; B: GG vs. AA; C: GA vs. AA; D: GG+GA vs. AA; E: GG vs. GA+AA. (DOCX) [file pone.0309747.s023.docx]

| A   | B   |
| --- | --- |
| C   | D   |
| E   |  |

**S23 Fig. Results of sensitivity analysis of rs4975616 in association with LC.**

A:G vs.A; B:GG vs.AA; C:GA vs.AA; D:GG+GA vs.AA; E:GG vs.GA+AA.
