# Supplementary material for: Differences in the risk association of TERT-CLPTM1L rs4975616 (A>G) with lung cancer between Caucasian and Asian populations: A meta-analysis
Source: PLoS One. 2024 Sep 10;19(9):e0309747. doi: 10.1371/journal.pone.0309747 (PMC11386447; doi:10.1371/journal.pone.0309747)
Supplement: S24 Fig — A: NSCLC; B: LUAD; C: LUSC; D: LC Smoking status. (DOCX) [file pone.0309747.s024.docx]

| A   | B   |
| --- | --- |
| C   | D   |

**S24 Fig. Results of sensitivity analysis of rs4975616(G vs.A) in association with LC of different ethnicity/pathological subtypes/smoking status.**

A:NSCLC; B:LUAD; C:LUSC; D:LC Smoking status.
