# Supplementary material for: Differences in the risk association of TERT-CLPTM1L rs4975616 (A>G) with lung cancer between Caucasian and Asian populations: A meta-analysis
Source: PLoS One. 2024 Sep 10;19(9):e0309747. doi: 10.1371/journal.pone.0309747 (PMC11386447; doi:10.1371/journal.pone.0309747)
Supplement: S26 Fig — A: NSCLC; B: LUAD; C: LUSC; D: LC Smokers; E: LC Non-smokers. (DOCX) [file pone.0309747.s026.docx]

| A  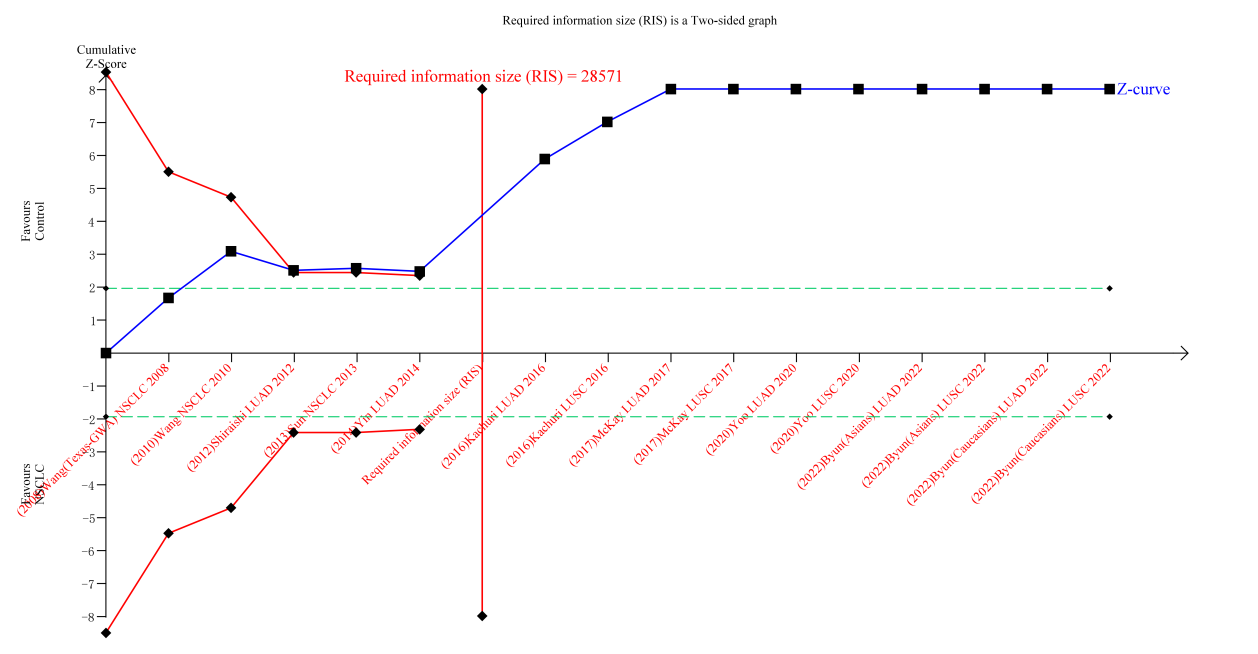 |
| --- |
| B  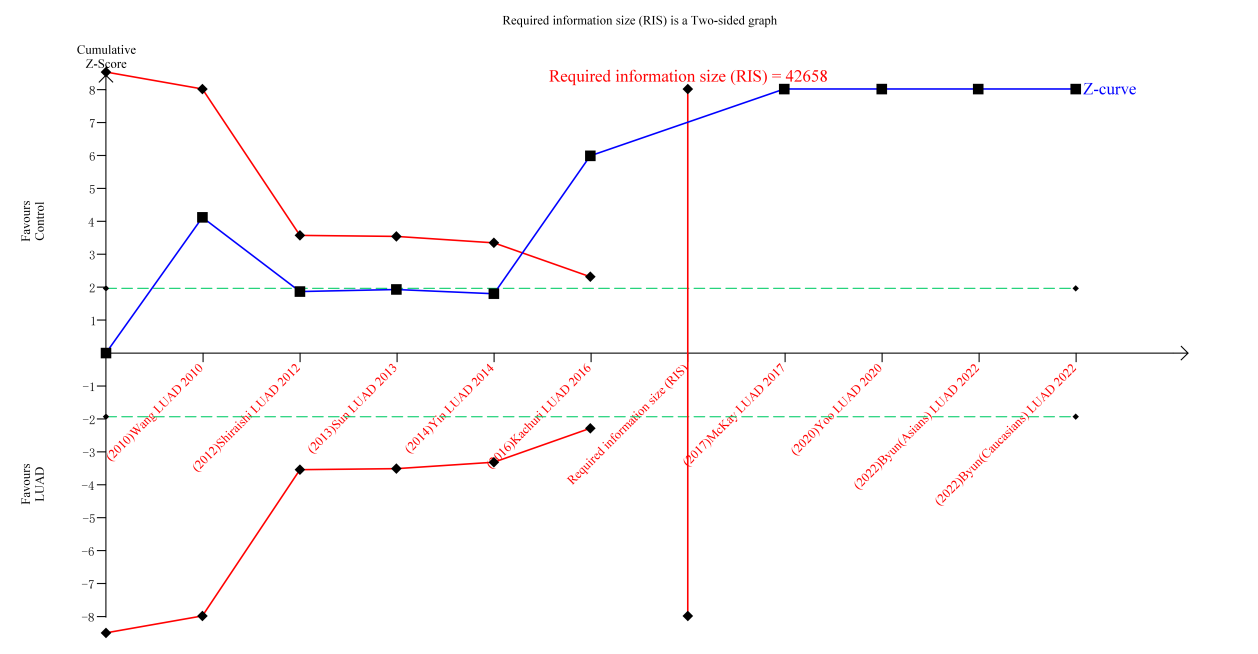 |
| C  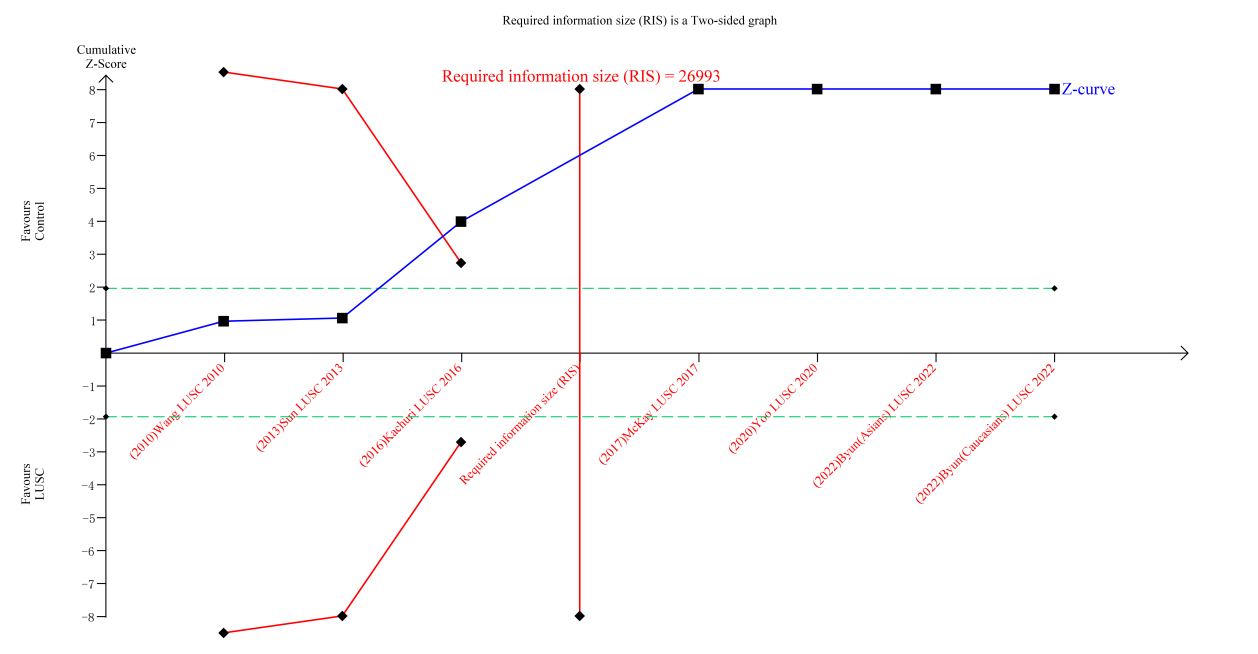 |
| D  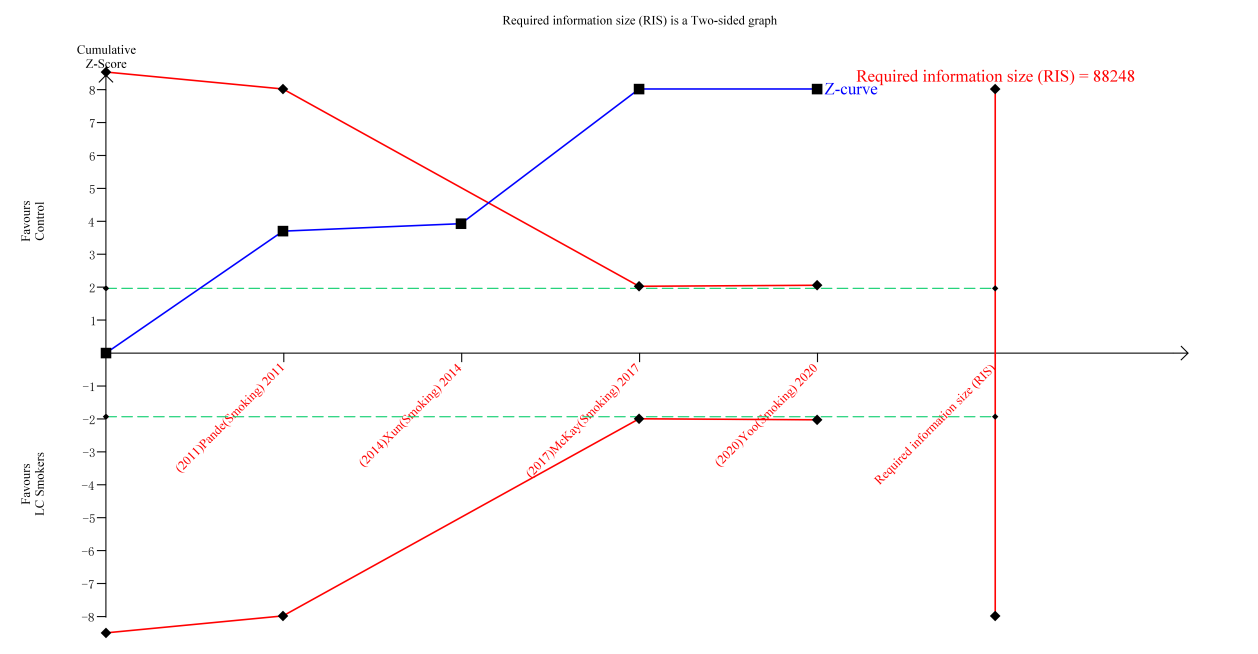 |
| E  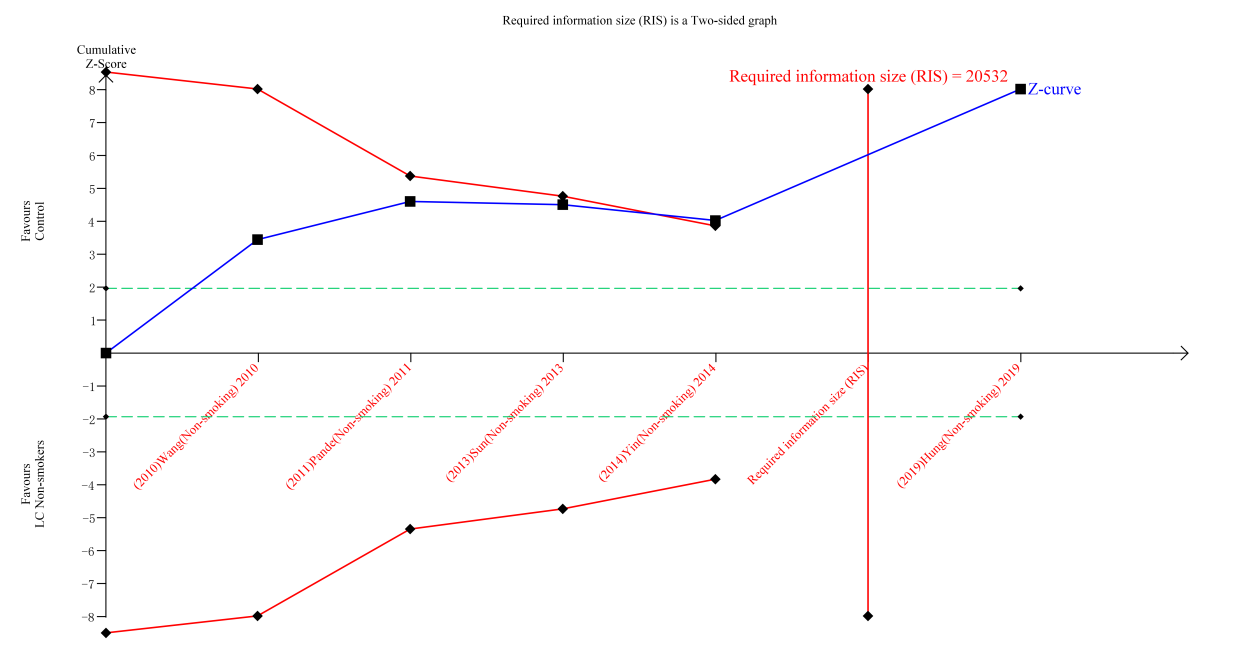 |

**S26 Fig. TSA results for the association of rs4975616(G vs.A) with LC in different pathological subtypes/smoking status.**

A:NSCLC; B: LUAD; C: LUSC; D:LC Smokers; E:LC Non-smokers.
