# Supplementary material for: Differences in the risk association of TERT-CLPTM1L rs4975616 (A>G) with lung cancer between Caucasian and Asian populations: A meta-analysis
Source: PLoS One. 2024 Sep 10;19(9):e0309747. doi: 10.1371/journal.pone.0309747 (PMC11386447; doi:10.1371/journal.pone.0309747)
Supplement: S1 Table — A: PubMed search strategy. B: A numbered table of all studies identified in the literature search. (DOCX) [file pone.0309747.s027.docx]

**S1 Table. Literature search strategy and search results.**

A: PubMed search strategy.

| Search number | Query | Search Details | Results |
| --- | --- | --- | --- |
| #6 | ((#1) AND (#2) OR (#3)OR (#4)AND (#5)) | ("lung cancer"[MeSH Terms] OR "LC"[tw] OR "lung cancer"[tw]) AND ("Telomerase"[MeSH Terms] OR "TERT"[tw] OR "telomerase reverse transcriptase"[tw] OR ("Cleft lip and cleft palate transmembrane protein 1"[MeSH Terms] OR "CLPTM1L"[tw] OR "Cleft lip and cleft palate transmembrane protein 1"[tw]OR "rs4975616"[MeSH Terms]) AND "polymorphism, genetic"[MeSH Terms] | 214 |
| #5 | polymorphism[MeSH Terms] | "polymorphism, genetic"[MeSH Terms] | 298,342 |
| #4 | rs4975616[MeSH Terms] | "rs4975616"[MeSH Terms] | 16,724 |
| #3 | (((Cleft lip and cleft palate transmembrane protein 1[MeSH Terms]) OR (CLPTM1L[tw])) OR (Cleft lip and cleft palate transmembrane protein 1[tw])) | "Cleft lip and cleft palate transmembrane protein 1"[MeSH Terms] OR "CLPTM1L"[tw] OR "Cleft lip and cleft palate transmembrane protein 1"[tw] | 34,768 |
| #2 | (((Telomerase reverse transcriptase[MeSH Terms]) OR (TERT[tw])) OR (Telomerase reverse transcriptase[tw])) | "Telomerase"[MeSH Terms] OR "TERT"[tw] OR "telomerase reverse transcriptase"[tw] | 43,486 |
| #1 | ((Lung cancer[MeSH Terms]) OR (LC[tw])) OR (Lung cancer[tw]) | "lung cancer"[MeSH Terms] OR "LC"[tw] OR "lung cancer"[tw] | 438,064 |

Note: Search Terms using MeSH terms and text Words.

Date: July 20, 2023

B: A numbered table of all studies identified in the literature search.

| Number | Authors | Include or exclude | Literatures |
| --- | --- | --- | --- |
| 1 | Ahn MJ 2012 | Excluded: Inconsistent research purpose | Ahn MJ, Won HH, Lee J, et al. The 18p11.22 locus is associated with never smoker non-small cell lung cancer susceptibility in Korean populations. Hum Genet. 2012;131(3):365-372. doi:10.1007/s00439-011-1080-z |
| 2 | Amos CI 2008 | Excluded: Study object discrepancy | Amos CI, Wu X, Broderick P, et al. Genome-wide association scan of tag SNPs identifies a susceptibility locus for lung cancer at 15q25.1. Nat Genet. 2008;40(5):616-622. doi:10.1038/ng.109 |
| 3 | Arimura-Omori M 2020 | Excluded: Study object discrepancy | Arimura-Omori M, Kiyohara C, Yanagihara T, et al. Association between Telomere-Related Polymorphisms and the Risk of IPF and COPD as a Precursor Lesion of Lung Cancer: Findings from the Fukuoka Tobacco-Related Lung Disease (FOLD) Registry. Asian Pac J Cancer Prev. 2020;21(3):667-673. Published 2020 Mar 1. doi:10.31557/APJCP.2020.21.3.667 |
| 4 | Azad AK 2014 | Excluded: Inconsistency in research methodology | Azad AK, Qiu X, Boyd K, et al. A genetic sequence variant (GSV) at susceptibility loci of 5p15.33 (TERT-CLPTM1L) is associated with survival outcome in locally advanced and metastatic non-small-cell lung cancer (NSCLC). Lung Cancer. 2014;84(3):289-294. doi:10.1016/j.lungcan.2014.03.008 |
| 5 | Aziz MA 2021 | Excluded: Literature review (meta) | Aziz MA, Jafrin S, Islam MS. Human TERT promoter polymorphism rs2853669 is associated with cancers: an updated meta-analysis. Hum Cell. 2021;34(4):1066-1081. doi:10.1007/s13577-021-00520-4 |
| 6 | Bae EY 2012 | Excluded: Inconsistent research purpose | Bae EY, Lee SY, Kang BK, et al. Replication of results of genome-wide association studies on lung cancer susceptibility loci in a Korean population. Respirology. 2012;17(4):699-706. doi:10.1111/j.1440-1843.2012.02165.x |
| 7 | Brenner DR 2013 | Excluded: Inconsistent research purpose | Brenner DR, Brennan P, Boffetta P, et al. Hierarchical modeling identifies novel lung cancer susceptibility variants in inflammation pathways among 10,140 cases and 11,012 controls [published correction appears in Hum Genet. 2016 Aug;135(8):963. doi: 10.1007/s00439-016-1692-4]. Hum Genet. 2013;132(5):579-589. doi:10.1007/s00439-013-1270-y |
| 8 | Broderick P 2009 | Included | Broderick P, Wang Y, Vijayakrishnan J, et al. Deciphering the impact of common genetic variation on lung cancer risk: a genome-wide association study. Cancer Res. 2009;69(16):6633-6641. doi:10.1158/0008-5472.CAN-09-0680 |
| 9 | Byun J 2022 | Included | Byun J, Han Y, Li Y, et al. Cross-ancestry genome-wide meta-analysis of 61,047 cases and 947,237 controls identifies new susceptibility loci contributing to lung cancer. Nat Genet. 2022;54(8):1167-1177. doi:10.1038/s41588-022-01115-x |
| 10 | Cao JL 2015 | Excluded: Literature review (meta) | Cao JL, Yuan P, Abuduwufuer A, Lv W, Yang YH, Hu J. Association between the TERT Genetic Polymorphism rs2853676 and Cancer Risk: Meta-Analysis of 76,108 Cases and 134,215 Controls. PLoS One. 2015;10(6):e0128829. Published 2015 Jun 4. doi:10.1371/journal.pone.0128829 |
| 11 | Chen XF 2012 | Excluded: Inconsistent research purpose | Chen XF, Cai S, Chen QG, et al. Multiple variants of TERT and CLPTM1L constitute risk factors for lung adenocarcinoma. Genet Mol Res. 2012;11(1):370-378. Published 2012 Feb 16. doi:10.4238/2012.February.16.2 |
| 12 | Chen Z 2017 | Excluded: Inconsistency in research methodology | Chen Z, Wang J, Bai Y, et al. The associations of TERT-CLPTM1L variants and TERT mRNA expression with the prognosis of early stage non-small cell lung cancer. Cancer Gene Ther. 2017;24(1):20-27. doi:10.1038/cgt.2016.74 |
| 13 | Cheng Y 2016 | Excluded: Inconsistent research purpose | Cheng Y, Jiang T, Zhu M, et al. Risk assessment models for genetic risk predictors of lung cancer using two-stage replication for Asian and European populations. Oncotarget. 2016;8(33):53959-53967. Published 2016 Jul 5. doi:10.18632/oncotarget.10403 |
| 14 | Cheng Y 2017 | Excluded: Study object discrepancy | Cheng Y, Wang C, Zhu M, et al. Targeted sequencing of chromosome 15q25 identified novel variants associated with risk of lung cancer and smoking behavior in Chinese. Carcinogenesis. 2017;38(5):552-558. doi:10.1093/carcin/bgx025 |
| 15 | Choi JE 2009 | Excluded: Inconsistent research purpose | Choi JE, Kang HG, Jang JS, et al. Polymorphisms in telomere maintenance genes and risk of lung cancer. Cancer Epidemiol Biomarkers Prev. 2009;18(10):2773-2781. doi:10.1158/1055-9965.EPI-09-0323 |
| 16 | Dai J 2019 | Excluded: Inconsistent research purpose | Dai J, Lv J, Zhu M, et al. Identification of risk loci and a polygenic risk score for lung cancer: a large-scale prospective cohort study in Chinese populations. Lancet Respir Med. 2019;7(10):881-891. doi:10.1016/S2213-2600(19)30144-4 |
| 17 | de Mello RA 2013 | Excluded: Inconsistent research purpose | de Mello RA, Ferreira M, Soares-Pires F, et al. The impact of polymorphic variations in the 5p15, 6p12, 6p21 and 15q25 Loci on the risk and prognosis of portuguese patients with non-small cell lung cancer. PLoS One. 2013;8(9):e72373. Published 2013 Sep 6. doi:10.1371/journal.pone.0072373 |
| 18 | Dong J 2017 | Excluded: Inconsistent research purpose | Dong J, Cheng Y, Zhu M, et al. Fine mapping of chromosome 5p15.33 identifies novel lung cancer susceptibility loci in Han Chinese. Int J Cancer. 2017;141(3):447-456. doi:10.1002/ijc.30702 |
| 19 | Dong Y 2015 | Excluded: Inconsistent research purpose | Dong Y, Chen J, Chen Z, et al. Evaluating the Association of Eight Polymorphisms with Cancer Susceptibility in a Han Chinese Population. PLoS One. 2015;10(7):e0132797. Published 2015 Jul 15. doi:10.1371/journal.pone.0132797 |
| 20 | Fachiroh J 2012 | Excluded: Study object discrepancy | Fachiroh J, Sangrajrang S, Johansson M, et al. Tobacco consumption and genetic susceptibility to nasopharyngeal carcinoma (NPC) in Thailand. Cancer Causes Control. 2012;23(12):1995-2002. doi:10.1007/s10552-012-0077-9 |
| 21 | Gao L 2014 | Excluded: Inconsistent research purpose | Gao L, Thakur A, Liang Y, et al. Polymorphisms in the TERT gene are associated with lung cancer risk in the Chinese Han population. Eur J Cancer Prev. 2014;23(6):497-501. doi:10.1097/CEJ.0000000000000086 |
| 22 | Hsiung CA 2010 | Excluded: Inconsistent research purpose | Hsiung CA, Lan Q, Hong YC, et al. The 5p15.33 locus is associated with risk of lung adenocarcinoma in never-smoking females in Asia. PLoS Genet. 2010;6(8):e1001051. Published 2010 Aug 5. doi:10.1371/journal.pgen.1001051 |
| 23 | Hu Z 2011 | Excluded: Inconsistent research purpose | Hu Z, Wu C, Shi Y, et al. A genome-wide association study identifies two new lung cancer susceptibility loci at 13q12.12 and 22q12.2 in Han Chinese. Nat Genet. 2011;43(8):792-796. Published 2011 Jul 3. doi:10.1038/ng.875 |
| 24 | Hung RJ 2019 | Included | Hung RJ, Spitz MR, Houlston RS, et al. Lung Cancer Risk in Never-Smokers of European Descent is Associated With Genetic Variation in the 5p15.33 TERT-CLPTM1Ll Region. J Thorac Oncol. 2019;14(8):1360-1369. doi:10.1016/j.jtho.2019.04.008 |
| 25 | Ito H 2012 | Excluded: Inconsistent research purpose | Ito H, McKay JD, Hosono S, et al. Association between a genome-wide association study-identified locus and the risk of lung cancer in Japanese population. J Thorac Oncol. 2012;7(5):790-798. doi:10.1097/JTO.0b013e3182475028 |
| 26 | Jaworowska E 2011 | Excluded: Inconsistent research purpose | Jaworowska E, Trubicka J, Lener MR, et al. Smoking related cancers and loci at chromosomes 15q25, 5p15, 6p22.1 and 6p21.33 in the Polish population. PLoS One. 2011;6(9):e25057. doi:10.1371/journal.pone.0025057 |
| 27 | Ji Z 2020 | Excluded: Inconsistent research purpose | Ji Z, Li Y, Xiang C, et al. TERT-rs33963617 and CLPTM1L-rs77518573 reduce the risk of non-small cell lung cancer in Chinese population. Gene. 2020;731:144357. doi:10.1016/j.gene.2020.144357 |
| 28 | Jiang M 2013 | Excluded: Inconsistent research purpose | Jiang M, Wu H, Qin C. Genetic variant rs401681 at 5p15.33 modifies susceptibility to lung cancer but not esophageal squamous cell carcinoma. PLoS One. 2013;8(12):e84277. Published 2013 Dec 30. doi:10.1371/journal.pone.0084277 |
| 29 | Jin G 2009 | Excluded: Inconsistent research purpose | Jin G, Xu L, Shu Y, et al. Common genetic variants on 5p15.33 contribute to risk of lung adenocarcinoma in a Chinese population. Carcinogenesis. 2009;30(6):987-990. doi:10.1093/carcin/bgp090 |
| 30 | Jin T 2016 | Included | Jin T, Li B, He N, et al. CLPTM1L polymorphism as a protective factor for lung cancer: a case-control study in southern Chinese population. Tumour Biol. 2016;37(8):10533-10538. doi:10.1007/s13277-016-4938-9 |
| 31 | Kachuri L 2016 | Included | Kachuri L, Amos CI, McKay JD, et al. Fine mapping of chromosome 5p15.33 based on a targeted deep sequencing and high density genotyping identifies novel lung cancer susceptibility loci. Carcinogenesis. 2016;37(1):96-105. doi:10.1093/carcin/bgv165 |
| 32 | Kachuri L 2019 | Excluded: Inconsistent research purpose | Kachuri L, Helby J, Bojesen SE, et al. Investigation of Leukocyte Telomere Length and Genetic Variants in Chromosome 5p15.33 as Prognostic Markers in Lung Cancer. Cancer Epidemiol Biomarkers Prev. 2019;28(7):1228-1237. doi:10.1158/1055-9965.EPI-18-1215 |
| 33 | Ke J 2013 | Excluded: Inconsistent research purpose | Ke J, Zhong R, Zhang T, et al. Replication study in Chinese population and meta-analysis supports association of the 5p15.33 locus with lung cancer. PLoS One. 2013;8(4):e62485. Published 2013 Apr 30. doi:10.1371/journal.pone.0062485 |
| 34 | Lan Q 2012 | Excluded: Inconsistent research purpose | Lan Q, Hsiung CA, Matsuo K, et al. Genome-wide association analysis identifies new lung cancer susceptibility loci in never-smoking women in Asia. Nat Genet. 2012;44(12):1330-1335. doi:10.1038/ng.2456 |
| 35 | Landi MT 2009 | Excluded: Inconsistent research purpose | Landi MT, Chatterjee N, Yu K, et al. A genome-wide association study of lung cancer identifies a region of chromosome 5p15 associated with risk for adenocarcinoma [published correction appears in Am J Hum Genet. 2011 Jun 10;88(6):861. doi: 10.1016/j.ajhg.2011.05.003]. Am J Hum Genet. 2009;85(5):679-691. doi:10.1016/j.ajhg.2009.09.012 |
| 36 | Lee DH 2017 | Excluded: Inconsistency in research methodology | Lee DH, Heo YR, Park WJ, Lee JH. A TERT-CLPTM1 locus polymorphism (rs401681) is associated with EGFR mutation in non-small cell lung cancer. Pathol Res Pract. 2017;213(11):1340-1343. doi:10.1016/j.prp.2017.09.028 |
| 37 | Li C 2013 | Excluded: Inconsistent research purpose | Li C, Yin Z, Wu W, Li X, Ren Y, Zhou B. Genetic variations in TERT-CLPTM1L genes and risk of lung cancer in Chinese women nonsmokers. PLoS One. 2013;8(5):e64988. Published 2013 May 30. doi:10.1371/journal.pone.0064988 |
| 38 | Li D 2015 | Excluded: Inconsistency in research methodology | Li D, Wei L, Xu B, et al. Association of GWAS-identified lung cancer susceptibility loci with survival length in patients with small-cell lung cancer treated with platinum-based chemotherapy [published correction appears in PLoS One. 2015 Mar 17;10(3):e0118689. doi: 10.1371/journal.pone.0118689]. PLoS One. 2014;9(11):e113574. Published 2014 Nov 21. doi:10.1371/journal.pone.0113574 |
| 39 | Li H 2012 | Excluded: Inconsistent research purpose | Li H, Yang L, Zhao X, et al. Prediction of lung cancer risk in a Chinese population using a multifactorial genetic model. BMC Med Genet. 2012;13:118. Published 2012 Dec 10. doi:10.1186/1471-2350-13-118 |
| 40 | Li X 2016 | Excluded: Inconsistent research purpose | Li X, Xu X, Fang J, et al. Rs2853677 modulates Snail1 binding to the TERT enhancer and affects lung adenocarcinoma susceptibility. Oncotarget. 2016;7(25):37825-37838. doi:10.18632/oncotarget.9339 |
| 41 | Liang Y 2014 | Included | Liang Y, Thakur A, Gao L, et al. Correlation of CLPTM1L polymorphisms with lung cancer susceptibility and response to cisplatin-based chemotherapy in a Chinese Han population. Tumour Biol. 2014;35(12):12075-12082. doi:10.1007/s13277-014-2508-6 |
| 42 | Liu C 2017 | Excluded: Literature review (meta) | Liu C, Cui H, Gu D, et al. Genetic polymorphisms and lung cancer risk: Evidence from meta-analyses and genome-wide association studies. Lung Cancer. 2017;113:18-29. doi:10.1016/j.lungcan.2017.08.026 |
| 43 | Liu P 2010 | Excluded: Study object discrepancy | Liu P, Vikis HG, Lu Y, et al. Cumulative effect of multiple loci on genetic susceptibility to familial lung cancer. Cancer Epidemiol Biomarkers Prev. 2010;19(2):517-524. doi:10.1158/1055-9965.EPI-09-0791 |
| 44 | Liu SG 2015 | Excluded: Inconsistent research purpose | Liu SG, Ma L, Cen QH, Huang JS, Zhang JX, Zhang JJ. Association of genetic polymorphisms in TERT-CLPTM1L with lung cancer in a Chinese population. Genet Mol Res. 2015;14(2):4469-4476. Published 2015 May 4. doi:10.4238/2015.May.4.4 |
| 45 | Liu Z 2018 | Excluded: Literature review (meta) | Liu Z, Wang T, Wu Z, et al. Association between TERT rs2853669 polymorphism and cancer risk: A meta-analysis of 9,157 cases and 11,073 controls. PLoS One. 2018;13(3):e0191560. Published 2018 Mar 13. doi:10.1371/journal.pone.0191560 |
| 46 | Lu X 2013 | Excluded: Inconsistent research purpose | Lu X, Ke J, Luo X, et al. The SNP rs402710 in 5p15.33 is associated with lung cancer risk: a replication study in Chinese population and a meta-analysis. PLoS One. 2013;8(10):e76252. Published 2013 Oct 23. doi:10.1371/journal.pone.0076252 |
| 47 | Luo X 2014 | Excluded: Inconsistent research purpose | Luo X, Lamsal LP, Xu WJ, et al. Genetic variant in CLPTM1L confers reduced risk of lung cancer: a replication study in Chinese and a meta-analysis. Asian Pac J Cancer Prev. 2014;15(21):9241-9247. doi:10.7314/apjcp.2014.15.21.9241 |
| 48 | Mandour I 2020 | Excluded: Inconsistent research purpose | Mandour I, Hussein SAM, Essam R, El-Hossainy MA. Study of genetic variants in chromosome 5p15.33 region in non-smoker lung cancer patients. Adv Respir Med. 2020;88(6):485-494. doi:10.5603/ARM.a2020.0161 |
| 49 | McKay JD 2008 | Included | McKay JD, Hung RJ, Gaborieau V, et al. Lung cancer susceptibility locus at 5p15.33. Nat Genet. 2008;40(12):1404-1406. doi:10.1038/ng.254 |
| 50 | McKay JD 2017 | Included | McKay JD, Hung RJ, Han Y, et al. Large-scale association analysis identifies new lung cancer susceptibility loci and heterogeneity in genetic susceptibility across histological subtypes. Nat Genet. 2017;49(7):1126-1132. doi:10.1038/ng.3892 |
| 51 | Miki D 2010 | Excluded: Inconsistent research purpose | Miki D, Kubo M, Takahashi A, et al. Variation in TP63 is associated with lung adenocarcinoma susceptibility in Japanese and Korean populations. Nat Genet. 2010;42(10):893-896. doi:10.1038/ng.667 |
| 52 | Mimouni A 2020 | Excluded: Inconsistent research purpose | Mimouni A, Rouleau E, Saulnier P, et al. Association of TERT, OGG1, and CHRNA5 Polymorphisms and the Predisposition to Lung Cancer in Eastern Algeria. Pulm Med. 2020;2020:7649038. Published 2020 Mar 20. doi:10.1155/2020/7649038 |
| 53 | Mirabello L 2010 | Excluded: Study object discrepancy | Mirabello L, Yu K, Kraft P, et al. The association of telomere length and genetic variation in telomere biology genes. Hum Mutat. 2010;31(9):1050-1058. doi:10.1002/humu.21314 |
| 54 | Myneni AA 2013 | Excluded: Inconsistent research purpose | Myneni AA, Chang SC, Niu R, et al. Genetic polymorphisms of TERT and CLPTM1L and risk of lung cancer--a case-control study in a Chinese population. Lung Cancer. 2013;80(2):131-137. doi:10.1016/j.lungcan.2013.01.021 |
| 55 | Pande M 2011 | Included | Pande M, Spitz MR, Wu X, Gorlov IP, Chen WV, Amos CI. Novel genetic variants in the chromosome 5p15.33 region associate with lung cancer risk. Carcinogenesis. 2011;32(10):1493-1499. doi:10.1093/carcin/bgr136 |
| 56 | Park SL 2014 | Excluded: Study object discrepancy | Park SL, Fesinmeyer MD, Timofeeva M, et al. Pleiotropic associations of risk variants identified for other cancers with lung cancer risk: the PAGE and TRICL consortia. J Natl Cancer Inst. 2014;106(4):dju061. doi:10.1093/jnci/dju061 |
| 57 | Pintarelli G 2017 | Excluded: Inconsistent research purpose | Pintarelli G, Cotroneo CE, Noci S, et al. Genetic susceptibility variants for lung cancer: replication study and assessment as expression quantitative trait loci. Sci Rep. 2017;7:42185. Published 2017 Feb 9. doi:10.1038/srep42185 |
| 58 | Rafnar T 2009 | Excluded: Inconsistent research purpose | Rafnar T, Sulem P, Stacey SN, et al. Sequence variants at the TERT-CLPTM1L locus associate with many cancer types. Nat Genet. 2009;41(2):221-227. doi:10.1038/ng.296 |
| 59 | Seow WJ 2017 | Excluded: Inconsistent research purpose | Seow WJ, Matsuo K, Hsiung CA, et al. Association between GWAS-identified lung adenocarcinoma susceptibility loci and EGFR mutations in never-smoking Asian women, and comparison with findings from Western populations. Hum Mol Genet. 2017;26(2):454-465. doi:10.1093/hmg/ddw414 |
| 60 | Shi J 2023 | Excluded: Inconsistent research purpose | Shi J, Shiraishi K, Choi J, et al. Genome-wide association study of lung adenocarcinoma in East Asia and comparison with a European population. Nat Commun. 2023;14(1):3043. Published 2023 May 26. doi:10.1038/s41467-023-38196-z |
| 61 | Shiraishi K 2012 | Included | Shiraishi K, Kunitoh H, Daigo Y, et al. A genome-wide association study identifies two new susceptibility loci for lung adenocarcinoma in the Japanese population. Nat Genet. 2012;44(8):900-903. Published 2012 Jul 15. doi:10.1038/ng.2353 |
| 62 | Snetselaar R 2018 | Excluded: Literature review (meta) | Snetselaar R, van Oosterhout MFM, Grutters JC, van Moorsel CHM. Telomerase Reverse Transcriptase Polymorphism rs2736100: A Balancing Act between Cancer and Non-Cancer Disease, a Meta-Analysis. Front Med (Lausanne). 2018;5:41. Published 2018 Feb 27. doi:10.3389/fmed.2018.00041 |
| 63 | Sun Y 2013 | Included | Sun Y, Zhang YJ, Kong XM. No association of XRCC1 and CLPTM1L polymorphisms with non-small cell lung cancer in a non-smoking Han Chinese population. Asian Pac J Cancer Prev. 2013;14(9):5171-5174. doi:10.7314/apjcp.2013.14.9.5171 |
| 64 | Tang J 2017 | Excluded: Literature review (meta) | Tang J, Hu C, Mei H, Peng L, Li H. CLPTM1L gene rs402710 (C > T) and rs401681 (C > T) polymorphisms associate with decreased cancer risk: a meta-analysis. Oncotarget. 2017;8(60):102446-102457. Published 2017 Nov 1. doi:10.18632/oncotarget.22268 |
| 65 | Thorgeirsson TE 2008 | Excluded: Study object discrepancy | Thorgeirsson TE, Geller F, Sulem P, et al. A variant associated with nicotine dependence, lung cancer and peripheral arterial disease. Nature. 2008;452(7187):638-642. doi:10.1038/nature06846 |
| 66 | Truong T 2010 | Excluded: Inconsistent research purpose | Truong T, Hung RJ, Amos CI, et al. Replication of lung cancer susceptibility loci at chromosomes 15q25, 5p15, and 6p21: a pooled analysis from the International Lung Cancer Consortium. J Natl Cancer Inst. 2010;102(13):959-971. doi:10.1093/jnci/djq178 |
| 67 | Tseng TS 2014 | Excluded: Inconsistent research purpose | Tseng TS, Park JY, Zabaleta J, et al. Role of nicotine dependence on the relationship between variants in the nicotinic receptor genes and risk of lung adenocarcinoma. PLoS One. 2014;9(9):e107268. Published 2014 Sep 18. doi:10.1371/journal.pone.0107268 |
| 68 | Wang H 2016 | Excluded: Inconsistent research purpose | Wang H , Yang H , Feng T ,et al.Effects of TERT gene polymorphism and environmental factor interactions on lung cancer risk in the Xi'an Han population.J Clin Exp Med 2016;9(2):4200-4210 |
| 69 | Wang H2013 | Excluded: Inconsistent research purpose | Wang H, Zhao Y, Ma J, et al. The genetic variant rs401681C/T is associated with the risk of non-small cell lung cancer in a Chinese mainland population. Genet Mol Res. 2013;12(1):67-73. Published 2013 Jan 22. doi:10.4238/2013.January.22.5 |
| 70 | Wang J 2017 | Excluded: Literature review (meta) | Wang J, Liu Q, Yuan S, et al. Genetic predisposition to lung cancer: comprehensive literature integration, meta-analysis, and multiple evidence assessment of candidate-gene association studies. Sci Rep. 2017;7(1):8371. Published 2017 Aug 21. doi:10.1038/s41598-017-07737-0 |
| 71 | Wang M 2020 | Excluded: Literature review (meta) | Wang M, Sun Y. Telomerase reverse transcriptase rs2736098 polymorphism is associated with lung cancer: A meta-analysis. J Int Med Res. 2020;48(10):300060520936173. doi:10.1177/0300060520936173IF: 1.6 Q4 |
| 72 | Wang Y 2008 | Included | Wang Y, Broderick P, Webb E, et al. Common 5p15.33 and 6p21.33 variants influence lung cancer risk. Nat Genet. 2008;40(12):1407-1409. doi:10.1038/ng.273 |
| 73 | Wang Y 2010 | Included | Wang Y, Broderick P, Matakidou A, Eisen T, Houlston RS. Role of 5p15.33 (TERT-CLPTM1L), 6p21.33 and 15q25.1 (CHRNA5-CHRNA3) variation and lung cancer risk in never-smokers. Carcinogenesis. 2010;31(2):234-238. doi:10.1093/carcin/bgp287 |
| 74 | Wang Z 2014 | Excluded: Inconsistent research purpose | Wang Z, Zhu B, Zhang M, et al. Imputation and subset-based association analysis across different cancer types identifies multiple independent risk loci in the TERT-CLPTM1L region on chromosome 5p15.33. Hum Mol Genet. 2014;23(24):6616-6633. doi:10.1093/hmg/ddu363 |
| 75 | Wauters E 2011 | Excluded: Inconsistent research purpose | Wauters E, Smeets D, Coolen J, et al. The TERT-CLPTM1L locus for lung cancer predisposes to bronchial obstruction and emphysema. Eur Respir J. 2011;38(4):924-931. doi:10.1183/09031936.00187110 |
| 76 | Wei R 2014 | Excluded: Study object discrepancy | Wei R, Li C, Zhang M, et al. Association between MUC5B and TERT polymorphisms and different interstitial lung disease phenotypes. Transl Res. 2014;163(5):494-502. doi:10.1016/j.trsl.2013.12.006 |
| 77 | Wu H 2013 | Excluded: Inconsistent research purpose | Wu H, Qiao N, Wang Y, et al. Association between the telomerase reverse transcriptase (TERT) rs2736098 polymorphism and cancer risk: evidence from a case-control study of non-small-cell lung cancer and a meta-analysis. PLoS One. 2013;8(11):e76372. Published 2013 Nov 19. doi:10.1371/journal.pone.0076372 |
| 78 | Xiao X 2017 | Excluded: Inconsistent research purpose | Xiao X, He W. Genetic polymorphisms in the TERT-CLPTM1L region and lung cancer susceptibility in Chinese males. Oncol Lett. 2017;14(2):1588-1594. doi:10.3892/ol.2017.6289 |
| 79 | Xing YL 2016 | Excluded: Inconsistent research purpose | Xing YL, Liu F, Li JF, et al. Case-Control Study on Impact of the Telomerase Reverse Transcriptase Gene Polymorphism and Additional Single Nucleotide Polymorphism (SNP)- SNP Interaction on Non-Small Cell Lung Cancers Risk in Chinese Han Population. J Clin Lab Anal. 2016;30(6):1071-1077. doi:10.1002/jcla.21982 |
| 80 | Xun WW 2011 | Excluded: Inconsistency in research methodology | Xun WW, Brennan P, Tjonneland A, et al. Single-nucleotide polymorphisms (5p15.33, 15q25.1, 6p22.1, 6q27 and 7p15.3) and lung cancer survival in the European Prospective Investigation into Cancer and Nutrition (EPIC). Mutagenesis. 2011;26(5):657-666. doi:10.1093/mutage/ger030 |
| 81 | Xun X 2014 | Included | Xun X, Wang H, Yang H, et al. CLPTM1L genetic polymorphisms and interaction with smoking and alcohol drinking in lung cancer risk: a case-control study in the Han population from northwest China. Medicine (Baltimore). 2014;93(28):e289. doi:10.1097/MD.0000000000000289 |
| 82 | Yang G 2010 | Excluded: Inconsistency in research methodology | Yang G, Li J, Zhang X, et al. Eimeria tenella: cloning and characterization of telomerase reverse transcriptase gene. Exp Parasitol. 2010;124(4):380-385. doi:10.1016/j.exppara.2009.12.004 |
| 83 | Yang P 2010 | Excluded: Inconsistent research purpose | Yang P, Li Y, Jiang R, et al. A rigorous and comprehensive validation: common genetic variations and lung cancer [published correction appears in Cancer Epidemiol Biomarkers Prev. 2010 Apr;19(4):1145. Li, Yan [added]]. Cancer Epidemiol Biomarkers Prev. 2010;19(1):240-244. doi:10.1158/1055-9965.EPI-09-0710 |
| 84 | Yang YC 2018 | Excluded: Inconsistency in research methodology | Yang YC, Fu WP, Zhang J, Zhong L, Cai SX, Sun C. rs401681 and rs402710 confer lung cancer susceptibility by regulating TERT expression instead of CLPTM1L in East Asian populations. Carcinogenesis. 2018;39(10):1216-1221. doi:10.1093/carcin/bgy084 |
| 85 | Yin Z 2014 | Included | Yin Z, Cui Z, Ren Y, et al. Genetic polymorphisms of TERT and CLPTM1L, cooking oil fume exposure, and risk of lung cancer: a case-control study in a Chinese non-smoking female population. Med Oncol. 2014;31(8):114. doi:10.1007/s12032-014-0114-5 |
| 86 | Yoo SS 2015 | Excluded: Inconsistent research purpose | Yoo SS, Do SK, Choi JE, et al. TERT Polymorphism rs2853669 Influences on Lung Cancer Risk in the Korean Population. J Korean Med Sci. 2015;30(10):1423-1428. doi:10.3346/jkms.2015.30.10.1423 |
| 87 | Yoo SS 2020 | Included | Yoo SS, Kang HG, Choi JE, et al. The effect of susceptibility variants, identified in never-smoking female lung cancer cases, on male smokers. Korean J Intern Med. 2020;35(4):929-935. doi:10.3904/kjim.2018.417 |
| 88 | Yoon KA 2010 | Excluded: Inconsistent research purpose | Yoon KA, Park JH, Han J, et al. A genome-wide association study reveals susceptibility variants for non-small cell lung cancer in the Korean population. Hum Mol Genet. 2010;19(24):4948-4954. doi:10.1093/hmg/ddq421 |
| 89 | Young RP 2011 | Excluded: Inconsistent research purpose | Young RP, Hopkins RJ, Whittington CF, Hay BA, Epton MJ, Gamble GD. Individual and cumulative effects of GWAS susceptibility loci in lung cancer: associations after sub-phenotyping for COPD. PLoS One. 2011;6(2):e16476. Published 2011 Feb 3. doi:10.1371/journal.pone.0016476 |
| 90 | Yuan P 2019 | Excluded: Inconsistency in research methodology | Yuan P, Huang S, Bao FC, et al. Discriminating association of a common telomerase reverse transcriptase promoter polymorphism with telomere parameters in non-small cell lung cancer with or without epidermal growth factor receptor mutation. Eur J Cancer. 2019;120:10-19. doi:10.1016/j.ejca.2019.06.024 |
| 91 | Zanetti KA 2016 | Excluded: Inconsistent research purpose | Zanetti KA, Wang Z, Aldrich M, et al. Genome-wide association study confirms lung cancer susceptibility loci on chromosomes 5p15 and 15q25 in an African-American population. Lung Cancer. 2016;98:33-42. doi:10.1016/j.lungcan.2016.05.008. |
| 92 | Zang Y 2014 | Excluded: Literature review (meta) | Zang Y, Nie W, Fang Z, Li B. Cleft lip and palate transmembrane protein 1 rs31489 polymorphism is associated with lung cancer risk: a meta-analysis. Tumour Biol. 2014;35(6):5583-5588. doi:10.1007/s13277-014-1736-0 |
| 93 | Zhang X 2022 | Excluded: Literature review (meta) | Zhang X, Chen Y, Yan D, Han J, Zhu L. TERT Gene rs2736100 and rs2736098 Polymorphisms are Associated with Increased Cancer Risk: A Meta-Analysis. Biochem Genet. 2022;60(1):241-266. doi:10.1007/s10528-021-10097-0 |
| 94 | Zhang Y 2014 | Excluded: Inconsistent research purpose | Zhang Y, Zhao M, Shen L, et al. Genetic polymorphisms of TERT and CLPTM1L and risk of lung cancer: a case-control study in northeast Chinese male population. Med Oncol. 2014;31(7):18. doi:10.1007/s12032-014-0018-4 |
| 95 | Zhao DP 2014 | Excluded: Literature review (meta) | Zhao DP, Yang CL, Zhou X, Ding JA, Jiang GN. Association between CLPTM1L polymorphisms (rs402710 and rs401681) and lung cancer susceptibility: evidence from 27 case-control studies. Mol Genet Genomics. 2014;289(5):1001-1012. doi:10.1007/s00438-014-0868-7 |
| 96 | Zhao MM 2014 | Excluded: Inconsistent research purpose | Zhao MM, Zhang Y, Shen L, et al. Genetic variations in TERT-CLPTM1L genes and risk of lung cancer in a Chinese population. Asian Pac J Cancer Prev. 2014;15(6):2809-2813. doi:10.7314/apjcp.2014.15.6.2809 |
| 97 | Zhao Z 2013 | Excluded: Inconsistent research purpose | Zhao Z, Li C, Yang L, et al. Significant association of 5p15.33 (TERT-CLPTM1L genes) with lung cancer in Chinese Han population. Exp Lung Res. 2013;39(2):91-98. doi:10.3109/01902148.2012.762436 |
| 98 | Zhong R 2013 | Excluded: Inconsistent research purpose | Zhong R, Liu L, Zou L, et al. Genetic variations in TERT-CLPTM1L locus are associated with risk of lung cancer in Chinese population. Mol Carcinog. 2013;52 Suppl 1:E118-E126. doi:10.1002/mc.22043 |
| 99 | Zhou M 2018 | Excluded: Literature review (meta) | Zhou M, Jiang B, Xiong M, Zhu X. Association Between TERT rs2736098 Polymorphisms and Cancer Risk-A Meta-Analysis. Front Physiol. 2018;9:377. Published 2018 Apr 11. doi:10.3389/fphys.2018.00377 |
| 100 | Zienolddiny S2009 | Excluded: Inconsistent research purpose | Zienolddiny S, Skaug V, Landvik NE, et al. The TERT-CLPTM1L lung cancer susceptibility variant associates with higher DNA adduct formation in the lung. Carcinogenesis. 2009;30(8):1368-1371. doi:10.1093/carcin/bgp131 |

Note: A total of 100 literatures were initially included after removing duplicates, 84 literatures were excluded after screening, and finally 16 literatures (includes 20 studies) were included in this Meta-analysis.
