# Supplementary material for: Differences in the risk association of TERT-CLPTM1L rs4975616 (A>G) with lung cancer between Caucasian and Asian populations: A meta-analysis
Source: PLoS One. 2024 Sep 10;19(9):e0309747. doi: 10.1371/journal.pone.0309747 (PMC11386447; doi:10.1371/journal.pone.0309747)
Supplement: S2 Table — (DOCX) [file pone.0309747.s028.docx]

**S2 Table. Basic features of the included study (1).**

| **ID** | **Studies** | **Country** | **Ethnicity** | **Type of studies** | **Type** | **LC(n)** | **Controls(n)** | **Gender (male %)** | | **Age (years)** | | **Percentage of smokers(%)** | |
| --- | --- | --- | --- | --- | --- | --- | --- | --- | --- | --- | --- | --- | --- |
|  |  |  |  |  |  |  |  | **LC** | **Controls** | **LC** | **Controls** | **LC** | **Controls** |
| 1 | Broderick (Phase 1) 2009[27] | UK | Caucasians | GWAS | LC | 1952 | 1438 | 60.56% | —— | 57 ± 6 | —— | Partial smoking | Partial smoking |
| 2 | Broderick (Phase 2) 2009[27] | UK | Caucasians | GWAS | LC | 2465 | 3005 | 68.56% | 49.82% | 72 ± 7 | 61 ± 11 | Partial smoking | Partial smoking |
| 3 | Byun (Caucasians) 2022[40] | European | Caucasians | GWAS | LC | 26683 | 25278 | —— | —— | —— | —— | No description | No description |
|  |  |  |  |  | LUAD | 9791 | 23173 |  |  |  |  |  |  |
|  |  |  |  |  | LUSC | 6107 | 23173 |  |  |  |  |  |  |
| 4 | Byun (Asians) 2022[40] | Asian | Asians | GWAS | LC | 7062 | 5372 | —— | —— | —— | —— | No description | No description |
|  |  |  |  |  | LUAD | 4630 | 5372 |  |  |  |  |  |  |
|  |  |  |  |  | LUSC | 1292 | 5372 |  |  |  |  |  |  |
| 5 | Hung 2019[37] | USA,Europe | Caucasians | GWAS | LC | 3636 | 6295 | 31.80% | 41.20% | 63.6 ± 12.4 | 61.9 ± 11.9 | Non-smoking | Non-smoking |
|  |  |  |  |  | LC Non-smoker | 3636 | 6295 |  |  |  |  |  |  |
| 6 | Jin 2016[28] | China | Asians | case-control study | LC | 554 | 696 | 75.10% | 56.30% | 58.17 ± 10.53 | 48.57 ± 9.47 | No description | No description |
| 7 | Kachuri 2016[11] | European,USA,Canada,Australia,Russia | Caucasians | case-control study | LC | 5164 | 5716 | 47.80% | 52.20% | 48.4% (>50) | 51.6% (>50) | 53.70% | 46.30% |
|  |  |  |  |  | LUAD | 1840 | 5716 |  |  |  |  |  |  |
|  |  |  |  |  | LUSC | 1296 | 5716 |  |  |  |  |  |  |
| 8 | Liang 2014[30] | China | Asians | case-control study | LC | 309 | 308 | 76.10% | 63.50% | 58 ± 10 | 50 ± 8 | 69.60% | 39.40% |
| 9 | McKay 2008[5] | USA,Europe | Caucasians | GWAS | LC | 2971 | 3746 | —— | —— | —— | —— | Partial smoking | Partial smoking |
| 10 | McKay 2017[39] | European | Caucasians | GWAS | LC | 29266 | 56450 | 62.00% | 53.00% | 88% (>50) | 88% (>50) | 91.00% | 69.00% |
|  |  |  |  |  | LUAD | 11273 | 55483 |  |  |  |  |  |  |
|  |  |  |  |  | LUSC | 7426 | 55627 |  |  |  |  |  |  |
|  |  |  |  |  | LC Smoker | 23223 | 16964 |  |  |  |  |  |  |
| 11 | Pande 2011[26] | USA | Caucasians | case-control study | LC | 1681 | 1235 | 59.50% | 40.50% | 63.5 ± 11 | 57.2 ± 13.2 | 72.52% | 58.87% |
|  |  |  |  |  | LC Smoker | 1219 | 727 |  |  |  |  |  |  |
|  |  |  |  |  | LC Non-smoker | 462 | 508 |  |  |  |  |  |  |
| 12 | Shiraishi 2012[31] | Japan | Asians | GWAS | LUAD | 1695 | 5333 | 52.40% | 61.30% | 58.8 ± 8.9 | 56.6 ± 14.2 | Partial smoking | Partial smoking |
| 13 | Sun 2013[32] | China | Asians | case-control study | NSCLC | 200 | 200 | 67.50% | 62.00% | 57.64(36-77) | 56.66 (33-80) | Non-smoking | Non-smoking |
|  |  |  |  |  | LUAD | 145 | 200 |  |  |  |  |  |  |
|  |  |  |  |  | LUSC | 55 | 200 |  |  |  |  |  |  |
|  |  |  |  |  | NSCLC Non-smoker | 200 | 200 |  |  |  |  |  |  |
|  |  |  |  |  | LUAD Non-smoker | 145 | 200 |  |  |  |  |  |  |
|  |  |  |  |  | LUSC Non-smoker | 55 | 200 |  |  |  |  |  |  |
| 14 | Wang(UK-GWA) 2008[25] | UK | Caucasians | GWAS | LC | 1947 | 1436 | 59.73% | —— | 62 ± 12 | —— | Partial smoking | Partial smoking |
| 15 | Wang(IARC-GWA) 2008[25] | European | Caucasians | GWAS | LC | 1923 | 2514 | —— | —— | —— | —— | Partial smoking | Partial smoking |
| 16 | Wang(Texas-GWA) 2008[25] | USA | Caucasians | GWAS | NSCLC | 1154 | 1137 | 57.07% | —— | 61.4% (≧60) | —— | Partial smoking | Partial smoking |
| 17 | Wang 2010[38] | UK | Caucasians | case-control study | LC | 239 | 553 | 57.74% | 18.99% | 67 (26-87) | 63（21-91） | Non-smoking | Non-smoking |
|  |  |  |  |  | NSCLC | 200 | 553 |  |  |  |  |  |  |
|  |  |  |  |  | SCLC | 39 | 553 |  |  |  |  |  |  |
|  |  |  |  |  | LUAD | 112 | 553 |  |  |  |  |  |  |
|  |  |  |  |  | LUSC | 48 | 553 |  |  |  |  |  |  |
|  |  |  |  |  | LC Non-smoker | 239 | 553 |  |  |  |  |  |  |
|  |  |  |  |  | NSCLC Non-smoker | 200 | 553 |  |  |  |  |  |  |
|  |  |  |  |  | SCLC Non-smoker | 39 | 553 |  |  |  |  |  |  |
|  |  |  |  |  | LUAD Non-smoker | 112 | 553 |  |  |  |  |  |  |
|  |  |  |  |  | LUSC Non-smoker | 48 | 553 |  |  |  |  |  |  |
| 18 | Xun 2014[33] | China | Asians | case-control study | LC | 228 | 301 | 78.10% | 62.50% | 58.7 ± 10 | 50.2 ± 8.1 | 72.40% | 30.20% |
|  |  |  |  |  | LC Smoker | 165 | 91 |  |  |  |  |  |  |
| 19 | Yin 2014[34] | China | Asians | case-control study | LC | 524 | 524 | 0% | 0% | 56.1 ± 11.9 | 56.8 ± 11.1 | Non-smoking | Non-smoking |
|  |  |  |  |  | LUAD | 365 | 524 |  |  |  |  |  |  |
|  |  |  |  |  | LC Non-smoker | 524 | 524 |  |  |  |  |  |  |
|  |  |  |  |  | LUAD Non-smoker | 365 | 524 |  |  |  |  |  |  |
| 20 | Yoo 2020[29] | Korea | Asians | case-control study | LC | 707 | 603 | 100% | 100% | 61.1 ± 8.0 | 60.6 ± 6.7 | 100% | 100% |
|  |  |  |  |  | SCLC | 118 | 603 |  |  |  |  |  |  |
|  |  |  |  |  | LUSC | 349 | 603 |  |  |  |  |  |  |
|  |  |  |  |  | LUAD | 209 | 603 |  |  |  |  |  |  |
|  |  |  |  |  | LC Smoker | 707 | 603 |  |  |  |  |  |  |
|  |  |  |  |  | SCLC Smoker | 118 | 603 |  |  |  |  |  |  |
|  |  |  |  |  | LUSC Smoker | 349 | 603 |  |  |  |  |  |  |
|  |  |  |  |  | LUAD Smoker | 209 | 603 |  |  |  |  |  |  |

LC:Lung cancer; NSCLC:non-small-cell lung carcinoma; SCLC:Small cell lung carcinoma; LUAD:Lung adenocarcinoma; LUSC:Lung squamous cell carcinoma.

Data are mean ± SD, or mean (IQR) ,or IQR, or n, unless otherwise stated

MISSING DATA: Blank as these data were not recorded in the original studies.
