# Supplementary material for: Differences in the risk association of TERT-CLPTM1L rs4975616 (A>G) with lung cancer between Caucasian and Asian populations: A meta-analysis
Source: PLoS One. 2024 Sep 10;19(9):e0309747. doi: 10.1371/journal.pone.0309747 (PMC11386447; doi:10.1371/journal.pone.0309747)
Supplement: S3 Table — (DOCX) [file pone.0309747.s029.docx]

**S3 Table. Basic features of the included study (2).**

| **ID** | **Studies** | **Type of studies** | **Genotyping methods** | **Type** | **LC(n)** | | | | | **Controls(n)** | | | | | **OR[95% Cl]** | **Hardy-**  **Weinberg** | **Name of data extractors and date of data extraction** | **Confirmation of inclusion criteria** |
| --- | --- | --- | --- | --- | --- | --- | --- | --- | --- | --- | --- | --- | --- | --- | --- | --- | --- | --- |
|  |  |  |  |  | **AA** | **AG** | **GG** | **A** | **G** | **AA** | **AG** | **GG** | **A** | **G** | **G vs.A** | **P_HWE_** |  |  |
| 1 | Broderick (Phase 1) 2009[27] | GWAS | Illumina Human BeadChips | LC | —— | —— | —— | —— | —— | —— | —— | —— | —— | —— | 0.86 [0.78, 0.95] | yes | X-ZW and WL.July 22, 2023 | Confirmation |
| 2 | Broderick (Phase 2) 2009[27] | GWAS | Illumina Infinium custom arrays | LC | —— | —— | —— | —— | —— | —— | —— | —— | —— | —— | 0.86 [0.79, 0.92] | yes | X-ZW and WL.July 22, 2023 | Confirmation |
| 3 | Byun (Caucasians) 2022[40] | GWAS | Oncoarray | LC | —— | —— | —— | 32903 | —— | —— | —— | —— | 29385 | —— | 0.86[0.84, 0.88] | yes | X-ZW and WL.July 28, 2023 | Confirmation |
|  |  |  |  | LUAD | —— | —— | —— | 12066 | —— | —— | —— | —— | 26904 | —— | 0.85[0.83, 0.88] |  |  |  |
|  |  |  |  | LUSC | —— | —— | —— | 7491 | —— | —— | —— | —— | 26868 | —— | 0.84[0.8, 0.87] |  |  |  |
| 4 | Byun (Asians) 2022[40] | GWAS | Oncoarray | LC | —— | —— | —— | 11585 | —— | —— | —— | —— | 8654 | —— | 0.91 [0.85, 0.97] | yes | X-ZW and WL.July 28, 2023 | Confirmation |
|  |  |  |  | LUAD | —— | —— | —— | 7600 | —— | —— | —— | —— | 8654 | —— | 0.89[0.83, 0.97] |  |  |  |
|  |  |  |  | LUSC | —— | —— | —— | 2121 | —— | —— | —— | —— | 8654 | —— | 0.87[0.78, 0.99] |  |  |  |
| 5 | Hung 2019[37] | GWAS | Illumina Infinium OmniExpress BeadChip +Illumina Human BeadChip+Illumina GoldenGate | LC | —— | —— | —— | —— | —— | —— | —— | —— | —— | —— | 0.78 [0.73, 0.83] | yes | X-ZW and WL.July 27, 2023 | Confirmation |
|  |  |  |  | LC Nonsmoker | —— | —— | —— | —— | —— | —— | —— | —— | —— | —— | 0.78 [0.73, 0.83] |  |  |  |
| 6 | Jin 2016[28] | case-control study | Sequenom Mass ARRAY | LC | —— | —— | —— | 970 | 139 | —— | —— | —— | 1178 | 214 | 0.78 [0.62, 0.99] | 0.561 | X-ZW and WL.July 27, 2023 | Confirmation |
| 7 | Kachuri 2016[11] | case-control study | Illumina HiSeq 2000 +Affymetrix Axiom | LC | —— | —— | —— | 6428 | 3900 | —— | —— | —— | 6642 | 4790 | 0.84 [0.79, 0.89] | 0.082 | X-ZW and WL.July 22, 2023 | Confirmation |
|  |  |  |  | LUAD | —— | —— | —— | 2289 | 1391 | —— | —— | —— | 6642 | 4790 | 0.79 [0.72, 0.86] |  |  |  |
|  |  |  |  | LUSC | —— | —— | —— | 1612 | 980 | —— | —— | —— | 6642 | 4790 | 0.84 [0.76, 0.93] |  |  |  |
| 8 | Liang 2014[30] | case-control study | Sequenom Mass ARRAY | LC | 228 | 77 | 4 | 533 | 85 | 217 | 83 | 8 | 517 | 99 | 0.83 [0.61, 1.14] | 0.999 | X-ZW and WL.July 22, 2023 | Confirmation |
| 9 | McKay 2008[5] | GWAS | Illumina chips | LC | —— | —— | —— | —— | —— | —— | —— | —— | —— | —— | 0.85 [0.79, 0.92] | yes | X-ZW and WL.July 28, 2023 | Confirmation |
| 10 | McKay 2017[39] | GWAS | OncoArray | LC | —— | —— | —— | —— | 23801 | —— | —— | —— | —— | —— | 0.87 [0.85, 0.90] | yes | X-ZW and WL.July 24, 2023 | Confirmation |
|  |  |  |  | LUAD | —— | —— | —— | —— | 9261 | —— | —— | —— | —— | —— | 0.86 [0.83, 0.89] |  |  |  |
|  |  |  |  | LUSC | —— | —— | —— | —— | 6108 | —— | —— | —— | —— | —— | 0.85 [0.82, 0.88] |  |  |  |
|  |  |  |  | LC Smoker | —— | —— | —— | —— | 18550 | —— | —— | —— | —— | —— | 0.89 [0.87, 0.92] |  |  |  |
| 11 | Pande 2011[26] | case-control study | Illumina iSelect Genotyping Beadchip | LC | —— | —— | —— | 2105 | 1257 | —— | —— | —— | 1398 | 1072 | 0.78 [0.70, 0.87] | 0.71 | X-ZW and WL.July 21, 2023 | Confirmation |
|  |  |  |  | LC Smoker | —— | —— | —— | —— | —— | —— | —— | —— | —— | —— | 0.78 [0.68, 0.89] | yes |  |  |
|  |  |  |  | LC Nonsmoker | —— | —— | —— | —— | —— | —— | —— | —— | —— | —— | 0.75 [0.62, 0.90] | yes |  |  |
| 12 | Shiraishi 2012[31] | GWAS | HumanOmni1-Quad+HumanOmniExpress | LUAD | —— | —— | —— | 2902 | 488 | —— | —— | —— | 9098 | 1568 | 0.98 [0.87, 1.09] | yes | X-ZW and WL.July 29, 2023 | Confirmation |
| 13 | Sun 2013[32] | case-control study | Mass ARRAY compact analyzer | NSCLC | 152 | 43 | 5 | 347 | 53 | 149 | 44 | 7 | 342 | 58 | 0.90 [0.60, 1.35] | 0.07 | X-ZW and WL.July 30, 2023 | Confirmation |
|  |  |  |  | LUAD | 109 | 33 | 3 | 251 | 39 | 149 | 44 | 7 | 342 | 58 | 0.92 [0.59, 1.42] |  |  |  |
|  |  |  |  | LUSC | 43 | 10 | 2 | 96 | 14 | 149 | 44 | 7 | 342 | 58 | 0.86 [0.46, 1.61] |  |  |  |
|  |  |  |  | NSCLC Nonsmoker | 152 | 43 | 5 | 347 | 53 | 149 | 44 | 7 | 342 | 58 | 0.90 [0.60, 1.35] |  |  |  |
|  |  |  |  | LUAD Non-smoker | 109 | 33 | 3 | 251 | 39 | 149 | 44 | 7 | 342 | 58 | 0.92 [0.59, 1.42] |  |  |  |
|  |  |  |  | LUSC Non-smoker | 43 | 10 | 2 | 96 | 14 | 149 | 44 | 7 | 342 | 58 | 0.86 [0.46, 1.61] |  |  |  |
| 14 | Wang(UK-GWA) 2008[25] | GWAS | Illumina HumanHap550 BeadChips | LC | 725 | 908 | 314 | 2358 | 1536 | 468 | 699 | 269 | 1635 | 1237 | 0.86 [0.78, 0.95] | 0.779 | X-ZW.July 25, 2023 | Confirmation |
| 15 | Wang(IARC-GWA) 2008[25] | GWAS | Illumina HumanHap300 Bead-Chip | LC | 800 | 902 | 221 | 2502 | 1344 | 971 | 1178 | 365 | 3120 | 1908 | 0.88 [0.80, 0.96] | 0.801 | X-ZW.July 25, 2023 | Confirmation |
| 16 | Wang(Texas-GWA) 2008[25] | GWAS | Illumina HumanHap300 Bead-Chip | NSCLC | 416 | 556 | 182 | 1388 | 920 | 367 | 579 | 191 | 1313 | 961 | 0.91 [0.81, 1.02] | 0.143 | X-ZW.July 25, 2023 | Confirmation |
| 17 | Wang 2010[38] | case-control study | Illumina Human550 BeadChip +Illumina Infinium | LC | 107 | 100 | 32 | 314 | 164 | 179 | 266 | 108 | 624 | 482 | 0.68 [0.54, 0.85] | 0.607 | X-ZW.July 25, 2023 | Confirmation |
|  |  |  |  | NSCLC | 91 | 84 | 25 | 266 | 134 | 179 | 266 | 108 | 624 | 482 | 0.65 [0.51, 0.83] |  |  |  |
|  |  |  |  | SCLC | 16 | 16 | 7 | 48 | 30 | 179 | 266 | 108 | 624 | 482 | 0.81 [0.51, 1.30] |  |  |  |
|  |  |  |  | LUAD | 59 | 42 | 11 | 160 | 64 | 179 | 266 | 108 | 624 | 482 | 0.52 [0.38, 0.71] |  |  |  |
|  |  |  |  | LUSC | 19 | 21 | 8 | 59 | 37 | 179 | 266 | 108 | 624 | 482 | 0.81 [0.53, 1.25] |  |  |  |
|  |  |  |  | LC Nonsmoker | 107 | 100 | 32 | 314 | 164 | 179 | 266 | 108 | 624 | 482 | 0.68 [0.54, 0.85] |  |  |  |
|  |  |  |  | NSCLC Non-smoker | 91 | 84 | 25 | 266 | 134 | 179 | 266 | 108 | 624 | 482 | 0.65 [0.51, 0.83] |  |  |  |
|  |  |  |  | SCLC Non-smoker | 16 | 16 | 7 | 48 | 30 | 179 | 266 | 108 | 624 | 482 | 0.81 [0.51, 1.30] |  |  |  |
|  |  |  |  | LUAD Non-smoker | 59 | 42 | 11 | 160 | 64 | 179 | 266 | 108 | 624 | 482 | 0.52 [0.38, 0.71] |  |  |  |
|  |  |  |  | LUSC Non-smoker | 19 | 21 | 8 | 59 | 37 | 179 | 266 | 108 | 624 | 482 | 0.81 [0.53, 1.25] |  |  |  |
| 18 | Xun 2014[33] | case-control study | Sequenom Mass ARRAY | LC | —— | —— | —— | 401 | 55 | —— | —— | —— | 506 | 96 | 0.74 [0.52, 1.06] | 0.84 | X-ZW and WL.July 24, 2023 | Confirmation |
|  |  |  |  | LC Smoker | —— | —— | —— | —— | —— | —— | —— | —— | —— | —— | 0.68 [0.41, 1.14] | 0.25 |  |  |
| 19 | Yin 2014[34] | case-control study | TaqMan | LC | 391 | 126 | 7 | 908 | 140 | 392 | 123 | 9 | 907 | 141 | 0.99 [0.77, 1.28] | 0.856 | X-ZW and WL.July 24, 2023 | Confirmation |
|  |  |  |  | LUAD | 271 | 90 | 4 | 632 | 98 | 392 | 123 | 9 | 907 | 141 | 1.00 [0.76, 1.32] |  |  |  |
|  |  |  |  | LC Nonsmoker | 391 | 126 | 7 | 908 | 140 | 392 | 123 | 9 | 907 | 141 | 0.99 [0.77, 1.28] |  |  |  |
|  |  |  |  | LUAD Non-smoker | 271 | 90 | 4 | 632 | 98 | 392 | 123 | 9 | 907 | 141 | 1.00 [0.76, 1.32] |  |  |  |
| 20 | Yoo 2020[29] | case-control study | Sequenom Mass ARRAY | LC | 541 | 154 | 12 | 1236 | 178 | 429 | 161 | 13 | 1019 | 187 | 0.78 [0.63, 0.98] | 0.642 | X-ZW.July 25, 2023 | Confirmation |
|  |  |  |  | SCLC | 86 | 28 | 4 | 200 | 36 | 429 | 161 | 13 | 1019 | 187 | 0.98 [0.67, 1.45] |  |  |  |
|  |  |  |  | LUSC | 268 | 75 | 6 | 611 | 87 | 429 | 161 | 13 | 1019 | 187 | 0.78 [0.59, 1.02] |  |  |  |
|  |  |  |  | LUAD | 162 | 46 | 1 | 370 | 48 | 429 | 161 | 13 | 1019 | 187 | 0.71 [0.50, 0.99] |  |  |  |
|  |  |  |  | LC Smoker | 541 | 154 | 12 | 1236 | 178 | 429 | 161 | 13 | 1019 | 187 | 0.78 [0.63, 0.98] |  |  |  |
|  |  |  |  | SCLC Smoker | 86 | 28 | 4 | 200 | 36 | 429 | 161 | 13 | 1019 | 187 | 0.98 [0.67, 1.45] |  |  |  |
|  |  |  |  | LUSC Smoker | 268 | 75 | 6 | 611 | 87 | 429 | 161 | 13 | 1019 | 187 | 0.78 [0.59, 1.02] |  |  |  |
|  |  |  |  | LUAD Smoker | 162 | 46 | 1 | 370 | 48 | 429 | 161 | 13 | 1019 | 187 | 0.71 [0.50, 0.99] |  |  |  |

LC:Lung cancer; NSCLC:non-small-cell lung carcinoma; LUAD:Lung adenocarcinoma; LUSC:Lung squamous cell carcinoma; PCR: Polymerase chain reaction; P_HWE_: P value of Hardy-Weinberg equilibrium.

Yes in P_HWE_: The original studies have been reported to be consistent with Hardy-Weinberg.

MISSING DATA: Since some of the original studies did not report complete genotype data, these were left blank and we only included the OR [95% Cl] of the allele models (G vs.A) reported in the original studies.
