# Supplementary material for: Differences in the risk association of TERT-CLPTM1L rs4975616 (A>G) with lung cancer between Caucasian and Asian populations: A meta-analysis
Source: PLoS One. 2024 Sep 10;19(9):e0309747. doi: 10.1371/journal.pone.0309747 (PMC11386447; doi:10.1371/journal.pone.0309747)
Supplement: S4 Table — (DOCX) [file pone.0309747.s030.docx]

**S4 Table. Newcastle Ottawa scale (NOS).**

|  |  | **Select** | | | | **Comparability^a^** | **Expose** | | | **Total score^b^** |
| --- | --- | --- | --- | --- | --- | --- | --- | --- | --- | --- |
| **ID** | **Studies** | **1** | **2** | **3** | **4** | **5** | **6** | **7** | **8** |  |
|  |  | **Ⅰ** | **Ⅱ** | **Ⅲ** | **Ⅳ** | **Ⅴ** | **Ⅵ** | **Ⅶ** | **Ⅷ** |  |
| 1 | Broderick (Phase 1) 2009[27] | ☆ | ☆ | ☆ |  | ☆☆ | ☆ | ☆ |  | 7☆ |
| 2 | Broderick (Phase 2) 2009[27] | ☆ | ☆ | ☆ | ☆ | ☆☆ | ☆ | ☆ |  | 8☆ |
| 3 | Byun (Caucasians) 2022[40] | ☆ | ☆ | ☆ | ☆ | ☆☆ | ☆ | ☆ |  | 8☆ |
| 4 | Byun (Asians) 2022[40] | ☆ | ☆ | ☆ | ☆ | ☆☆ | ☆ | ☆ |  | 8☆ |
| 5 | Hung 2019[37] | ☆ | ☆ | ☆ | ☆ | ☆☆ | ☆ | ☆ |  | 8☆ |
| 6 | Jin 2016[28] | ☆ | ☆ | ☆ | ☆ | ☆☆ |  | ☆ |  | 7☆ |
| 7 | Kachuri 2016[11] | ☆ | ☆ | ☆ | ☆ | ☆☆ | ☆ | ☆ |  | 8☆ |
| 8 | Liang 2014[30] | ☆ | ☆ | ☆ | ☆ | ☆☆ | ☆ | ☆ |  | 8☆ |
| 9 | McKay 2008[5] | ☆ | ☆ | ☆ | ☆ | ☆☆ | ☆ | ☆ |  | 8☆ |
| 10 | McKay 2017[39] | ☆ | ☆ | ☆ | ☆ | ☆☆ | ☆ | ☆ |  | 8☆ |
| 11 | Pande 2011[26] | ☆ | ☆ | ☆ | ☆ | ☆☆ | ☆ | ☆ |  | 8☆ |
| 12 | Shiraishi 2012[31] | ☆ | ☆ | ☆ | ☆ | ☆☆ | ☆ | ☆ |  | 8☆ |
| 13 | Sun 2013[32] | ☆ | ☆ | ☆ | ☆ | ☆☆ | ☆ | ☆ |  | 8☆ |
| 14 | Wang(UK-GWA) 2008[25] | ☆ | ☆ | ☆ | ☆ | ☆☆ | ☆ | ☆ |  | 8☆ |
| 15 | Wang(Texas-GWA) 2008[25] | ☆ | ☆ | ☆ | ☆ | ☆☆ | ☆ | ☆ |  | 8☆ |
| 16 | Wang(IARC-GWA) 2008[25] | ☆ | ☆ | ☆ | ☆ | ☆☆ | ☆ | ☆ |  | 8☆ |
| 17 | Wang 2010[38] | ☆ | ☆ | ☆ | ☆ | ☆☆ | ☆ | ☆ |  | 8☆ |
| 18 | Xun 2014[33] | ☆ | ☆ | ☆ | ☆ | ☆☆ | ☆ | ☆ |  | 8☆ |
| 19 | Yin 2014[34] | ☆ | ☆ | ☆ | ☆ | ☆☆ | ☆ | ☆ |  | 8☆ |
| 20 | Yoo 2020[29] | ☆ | ☆ | ☆ | ☆ | ☆☆ | ☆ | ☆ |  | 8☆ |

Note: a: Two stars with the highest comparability;b: Full score is 9☆.1-8:Case-control studies (CC);Ⅰ-Ⅷ:Cohort studies(CS).

1. Case definition;2: Case manifestations;3. Selection of control group;4. Definition of control group;5: Choose the most important/second most important factor;6. Determination of exposure;7. Methods for determining cases and control groups; 8: No response rate.

I: representativeness of exposure; II: selection of non exposed persons; III: Determination of exposure; IV: proof of no interesting results at the beginning; V: comparability; VI: evaluation of results; VII: long enough follow-up time; VIII: adequacy of follow-up.
