# Supplementary material for: Differences in the risk association of TERT-CLPTM1L rs4975616 (A>G) with lung cancer between Caucasian and Asian populations: A meta-analysis
Source: PLoS One. 2024 Sep 10;19(9):e0309747. doi: 10.1371/journal.pone.0309747 (PMC11386447; doi:10.1371/journal.pone.0309747)
Supplement: S5 Table — (DOCX) [file pone.0309747.s031.docx]

**S5 Table. The LC results of additive, heterozygous, dominant and recessive genetic models in rs4975616.**

| **Genetic models** | **Subgroups** | **Studies (n)** | **Heterogeneity test** | | **Sample** | | **Model** | **OR[95% CI]** | **Effect**  **p value** | **Subgroup differences** | | **Publication bias** | | **OR[95% Pl]** |
| --- | --- | --- | --- | --- | --- | --- | --- | --- | --- | --- | --- | --- | --- | --- |
|  |  |  | **P values** | **I^2^(%)** | **Cases**  **(n)** | **Controls**  **(n)** |  |  |  | **P values** | **I^2^(%)** | **P_Begg_** | **P_Egger_** |  |
| **GG vs.AA** | Caucasians | 4 | 0.27 | 24 | 2797 | 2918 | Random | **0.74 [0.64, 0.85]** | **＜0.0001** | **——** | **——** | 1.000 | 0.292 | 0.74 [0.48, 1.14] |
|  | Asians | 4 | 0.93 | 0 | 1340 | 1224 | Random | 0.69 [0.42, 1.13] | 0.14 | **——** | **——** | 0.174 | 0.379 | 0.69 [0.42, 1.13] |
|  | Overall | 8 | 0.72 | 0 | 4137 | 4142 | Random | **0.74 [0.66, 0.83]** | **＜0.00001** | 0.77 | 0 | 0.337 | 0.091 | 0.74 [0.66, 0.83] |
| **GA vs.AA** | Caucasians | 4 | 0.17 | 40 | 4514 | 4707 | Random | **0.85 [0.76, 0.95]** | **0.004** | **——** | **——** | 0.174 | 0.042 | 0.85 [0.57, 1.26] |
|  | Asians | 4 | 0.47 | 0 | 1712 | 1598 | Random | 0.88 [0.75, 1.03] | 0.11 | **——** | **——** | 0.497 | 0.605 | 0.88 [0.75, 1.03] |
|  | Overall | 8 | 0.37 | 8 | 6226 | 6305 | Random | **0.86 [0.80, 0.93]** | **0.0003** | 0.70 | 0 | 0.631 | 0.000 | 0.86 [0.76, 0.98] |
| **GG+GA vs.AA** | Caucasians | 4 | 0.13 | 48 | 5263 | 5640 | Random | **0.82 [0.73, 0.92]** | **0.0006** | **——** | **——** | 0.174 | 0.065 | 0.82 [0.53, 1.26] |
|  | Asians | 4 | 0.49 | 0 | 1740 | 1635 | Random | 0.86 [0.74, 1.01] | 0.06 | **——** | **——** | 0.497 | 0.676 | 0.86 [0.74, 1.01] |
|  | Overall | 8 | 0.31 | 16 | 7003 | 7275 | Random | **0.84 [0.77, 0.91]** | **＜0.0001** | 0.56 | 0 | 0.631 | 0.001 | 0.84 [0.72, 0.97] |
| **GG vs.GA+AA** | Caucasians | 4 | 0.37 | 4 | 5263 | 5640 | Random | **0.81 [0.73, 0.91]** | **0.0003** | **——** | **——** | 1.000 | 0.565 | 0.82 [0.63, 1.06] |
|  | Asians | 4 | 0.93 | 0 | 1740 | 1635 | Random | 0.71 [0.43, 1.17] | 0.18 | **——** | **——** | 0.042 | 0.257 | 0.71 [0.43, 1.17] |
|  | Overall | 8 | 0.79 | 0 | 7003 | 7275 | Random | **0.81 [0.73, 0.90]** | **＜0.0001** | 0.59 | 0 | 0.15 | 0.087 | 0.81 [0.73, 0.90] |
