# Supplementary material for: Differences in the risk association of TERT-CLPTM1L rs4975616 (A>G) with lung cancer between Caucasian and Asian populations: A meta-analysis
Source: PLoS One. 2024 Sep 10;19(9):e0309747. doi: 10.1371/journal.pone.0309747 (PMC11386447; doi:10.1371/journal.pone.0309747)
Supplement: S6 Table — (DOCX) [file pone.0309747.s032.docx]

**S6 Table. The publication bias and 95% Prediction Interval for the association of rs4975616(G vs.A) with LC of different smoking status.**

| Smoking status | n | Begg's | | Begg's | | cont.  corr. | | Egger's | | OR[95% PI] |
| --- | --- | --- | --- | --- | --- | --- | --- | --- | --- | --- |
|  |  | score | s.d. | z | p | z | p | bias | p |  |
| Non-smokers | 5 | 2 | 4.082 | 0.49 | 0.624 | 0.24 | 0.806 | 0.45 | 0.681 | 0.79 [0.61, 1.01] |
| Smokers | 4 | 0 | 2.944 | 0 | 1 | -0.34 | 1 | -1.53 | 0.073 | 0.83 [0.57, 1.22] |
| overall | 9 | 2 | 5.033 | 0.4 | 0.691 | 0.2 | 0.843 | -1.21 | 0.003 | 0.81 [0.64, 1.01] |
