# Supplementary material for: Differences in the risk association of TERT-CLPTM1L rs4975616 (A>G) with lung cancer between Caucasian and Asian populations: A meta-analysis
Source: PLoS One. 2024 Sep 10;19(9):e0309747. doi: 10.1371/journal.pone.0309747 (PMC11386447; doi:10.1371/journal.pone.0309747)
Supplement: S7 Table — (DOCX) [file pone.0309747.s033.docx]

**S7 Table. The results of sensitivity analysis.**

| **Study omitted** | **Year** | **Estimate** | **[95% Conf. Interval]** | |
| --- | --- | --- | --- | --- |
| **a. G vs.A (LC)** | | | | |
| Broderick(Phase 1) | 2009 | -0.15520385 | -0.18015611 | -0.13025159 |
| Broderick(Phase 2) | 2009 | -0.15504308 | -0.18037143 | -0.12971473 |
| Byun(Asians) | 2022 | -0.1595059 | -0.18382247 | -0.13518934 |
| Byun(Caucasians) | 2022 | -0.15646192 | -0.18570662 | -0.12721722 |
| Hung | 2019 | -0.14502801 | -0.16362645 | -0.12642957 |
| Jin | 2016 | -0.15397458 | -0.17802206 | -0.1299271 |
| Kachuri | 2016 | -0.15321006 | -0.17863907 | -0.12778105 |
| Liang | 2014 | -0.15478869 | -0.17904077 | -0.1305366 |
| McKay | 2008 | -0.15489295 | -0.18033782 | -0.12944809 |
| McKay | 2017 | -0.15887925 | -0.1868892 | -0.13086928 |
| Pande | 2011 | -0.15090601 | -0.17406872 | -0.12774329 |
| Shiraishi | 2012 | -0.15859924 | -0.18077478 | -0.13642372 |
| Sun | 2013 | -0.15511476 | -0.17933032 | -0.13089921 |
| Wang | 2010 | -0.15206026 | -0.17424269 | -0.12987782 |
| Wang(IARC-GWA) | 2008 | -0.15622799 | -0.18120909 | -0.13124689 |
| Wang(Texas-GWA) | 2008 | -0.15702395 | -0.18136764 | -0.13268027 |
| Wang(UK-GWA) | 2008 | -0.15520385 | -0.18015611 | -0.13025159 |
| Xun | 2014 | -0.15422398 | -0.17817037 | -0.13027757 |
| Yin | 2014 | -0.15601501 | -0.17977768 | -0.13225235 |
| Yoo | 2020 | -0.15385517 | -0.17788672 | -0.12982361 |
| Combined |  | -0.15480311 | -0.17853248 | -0.13107373 |
| **b.GG vs.AA (LC)** | | | | |
| Liang | 2014 | -0.29627362 | -0.40566161 | -0.18688563 |
| Sun | 2013 | -0.29918641 | -0.40863201 | -0.18974081 |
| Wang(UK-GWA) | 2008 | -0.30554587 | -0.43844208 | -0.17264965 |
| Wang(Texas-GWA) | 2008 | -0.33186245 | -0.45400998 | -0.20971492 |
| Wang(IARC-GWA) | 2008 | -0.29094717 | -0.4280422 | -0.15385213 |
| Wang | 2010 | -0.27813765 | -0.39004281 | -0.16623247 |
| Yin | 2014 | -0.30032471 | -0.40995103 | -0.1906984 |
| Yoo | 2020 | -0.2994082 | -0.40940842 | -0.18940797 |
| Combined |  | -0.29969617 | -0.40865568 | -0.19073667 |
| **c.GA vs.AA (LC)** | | | | |
| Liang | 2014 | -0.14935336 | -0.23814708 | -0.06055963 |
| Sun | 2013 | -0.15066962 | -0.23660314 | -0.06473609 |
| Wang(UK-GWA) | 2008 | -0.14145692 | -0.24020898 | -0.04270486 |
| Wang(Texas-GWA) | 2008 | -0.14594264 | -0.24273689 | -0.04914839 |
| Wang(IARC-GWA) | 2008 | -0.17749515 | -0.2681638 | -0.08682652 |
| Wang | 2010 | -0.12557055 | -0.20095453 | -0.05018659 |
| Yin | 2014 | -0.15334751 | -0.22938029 | -0.07731473 |
| Yoo | 2020 | -0.13068914 | -0.210783 | -0.0505953 |
| Combined |  | -0.14396515 | -0.2214877 | -0.06644261 |
| **d.GG+GA vs.AA (LC)** | | | | |
| Liang | 2014 | -0.18433054 | -0.27287495 | -0.09578612 |
| Sun | 2013 | -0.186267 | -0.27233326 | -0.10020075 |
| Wang(UK-GWA) | 2008 | -0.17532985 | -0.27663806 | -0.07402163 |
| Wang(Texas-GWA) | 2008 | -0.18780784 | -0.28438166 | -0.09123405 |
| Wang(IARC-GWA) | 2008 | -0.2030845 | -0.30005938 | -0.10610963 |
| Wang | 2010 | -0.16049525 | -0.23144941 | -0.08954111 |
| Yin | 2014 | -0.19160089 | -0.26582956 | -0.11737221 |
| Yoo | 2020 | -0.17287304 | -0.25938681 | -0.08635926 |
| Combined |  | -0.18099543 | -0.2592138 | -0.10277705 |
| **e.GG vs.GA+AA (LC)** | | | | |
| Liang | 2014 | -0.21284325 | -0.31467444 | -0.11101207 |
| Sun | 2013 | -0.21556734 | -0.31740639 | -0.1137283 |
| Wang(UK-GWA) | 2008 | -0.2331392 | -0.359451 | -0.10682739 |
| Wang(Texas-GWA) | 2008 | -0.25184706 | -0.36507323 | -0.13862091 |
| Wang(IARC-GWA) | 2008 | -0.18557259 | -0.31125826 | -0.05988692 |
| Wang | 2010 | -0.20236559 | -0.30690005 | -0.09783112 |
| Yin | 2014 | -0.21602929 | -0.31803793 | -0.11402063 |
| Yoo | 2020 | -0.21599822 | -0.31827763 | -0.11371882 |
| Combined |  | -0.2165191 | -0.31797561 | -0.11506258 |
| **f.G vs.A (NSCLC)** | | | | |
| Byun(Asians) LUAD | 2022 | -0.1604466 | -0.18500254 | -0.13589066 |
| Byun(Asians) LUSC | 2022 | -0.15791483 | -0.18288729 | -0.13294238 |
| Byun(Caucasians) LUAD | 2022 | -0.15665606 | -0.18635817 | -0.12695394 |
| Byun(Caucasians) LUSC | 2022 | -0.15343964 | -0.18014951 | -0.12672979 |
| Kachuri LUAD | 2016 | -0.1530648 | -0.17523189 | -0.13089773 |
| Kachuri LUSC | 2016 | -0.15619463 | -0.18144907 | -0.13094017 |
| McKay LUAD | 2017 | -0.15793799 | -0.18693776 | -0.12893821 |
| McKay LUSC | 2017 | -0.1554455 | -0.18378171 | -0.12710927 |
| Shiraishi LUAD | 2012 | -0.16153857 | -0.18037829 | -0.14269887 |
| Sun NSCLC | 2013 | -0.15724279 | -0.18173887 | -0.13274673 |
| Wang NSCLC | 2010 | -0.15590324 | -0.17573819 | -0.13606828 |
| Wang(Texas-GWA) NSCLC | 2008 | -0.15951154 | -0.18362986 | -0.13539323 |
| Yin LUAD | 2014 | -0.15832336 | -0.18182682 | -0.13481991 |
| Yoo LUAD | 2020 | -0.15629476 | -0.17986305 | -0.13272648 |
| Yoo LUSC | 2020 | -0.15636522 | -0.18058506 | -0.13214538 |
| Combined |  | -0.15716795 | -0.18081618 | -0.13351972 |
| **g.G vs.A (LUAD)** | | | | |
| Byun(Asians) LUAD | 2022 | -0.16379237 | -0.22042428 | -0.10716046 |
| Byun(Caucasians) LUAD | 2022 | -0.16147991 | -0.23932424 | -0.08363558 |
| Kachuri LUAD | 2016 | -0.14040488 | -0.19195415 | -0.0888556 |
| McKay LUAD | 2017 | -0.16264896 | -0.23966266 | -0.08563524 |
| Shiraishi LUAD | 2012 | -0.16946544 | -0.21679738 | -0.1221335 |
| Sun LUAD | 2013 | -0.15602823 | -0.20691 | -0.10514644 |
| Wang LUAD | 2010 | -0.14655416 | -0.18333586 | -0.10977244 |
| Yin LUAD | 2014 | -0.15954812 | -0.20963813 | -0.10945809 |
| Yoo LUAD | 2020 | -0.15108795 | -0.20084928 | -0.10132664 |
| Combined |  | -0.15494463 | -0.2043851 | -0.10550416 |
| **h.G vs.A (LUSC)** | | | | |
| Byun(Asians) LUSC | 2022 | -0.17155519 | -0.19884399 | -0.14426641 |
| Byun(Caucasians) LUSC | 2022 | -0.16450132 | -0.19778062 | -0.13122202 |
| Kachuri LUSC | 2016 | -0.1694124 | -0.19699462 | -0.14183018 |
| McKay LUSC | 2017 | -0.17510307 | -0.21292596 | -0.13728017 |
| Sun LUSC | 2013 | -0.16973183 | -0.1963626 | -0.14310107 |
| Wang LUSC | 2010 | -0.16955663 | -0.19621469 | -0.14289859 |
| Yoo LUSC | 2020 | -0.16893548 | -0.1956702 | -0.14220075 |
| Combined |  | -0.16969641 | -0.19630315 | -0.14308968 |
| **i.G vs.A (LC Smoking status)** | | | | |
| Hung(Non-smoking) | 2019 | -0.20552462 | -0.29828936 | -0.1127599 |
| McKay(Smoking) | 2017 | -0.24998543 | -0.30019489 | -0.19977596 |
| Pande(Non-smoking) | 2011 | -0.20588891 | -0.29340246 | -0.11837536 |
| Pande(Smoking) | 2011 | -0.20959581 | -0.301135 | -0.11805663 |
| Sun(Non-smoking) | 2013 | -0.21980065 | -0.30575418 | -0.13384713 |
| Wang(Non-smoking) | 2010 | -0.19831049 | -0.28050405 | -0.11611695 |
| Xun(Smoking) | 2014 | -0.2112731 | -0.29546344 | -0.12708277 |
| Yin(Non-smoking) | 2014 | -0.23194218 | -0.31852826 | -0.14535607 |
| Yoo(Smoking) | 2020 | -0.21285313 | -0.30123803 | -0.12446825 |
| Combined |  | -0.2151379 | -0.2977619 | -0.13251389 |
